# Thermodynamic control of Cu-I nanotopologies enables solar-driven CO_2_-to-multicarbon conversion

**DOI:** 10.1126/sciadv.aec6434

**Published:** 2026-07-17

**Authors:** Qixing Zhang, Jing Gao, Muchen Li, Ying Yang, Guanlan Chen, Han He, Xingfei Chen, Xiaobing Gu, Yajun Gao, Yongcai He, Bo He, Xixiang Xu, Zhenguo Li, Ying Zhao, Lijun Zhang, Michael Grätzel, Xiaodan Zhang

**Affiliations:** ^1^ Institute of Photoelectronic Thin Film Devices and Technology, Renewable Energy Conversion and Storage Center, Nankai University, Tianjin 300350, P. R. China.; ^2^ State Key Laboratory of Photovoltaic Materials and Cells, Nankai University, Tianjin 300350, P. R. China.; ^3^ Tianjin Key Laboratory of Efficient Utilization of Solar Energy, Tianjin 300350, P. R. China.; ^4^ Haihe Laboratory of Sustainable Chemical Transformations, Tianjin 300192, P. R. China.; ^5^ Engineering Research Center of Thin Film Photoelectronic Technology of Ministry of Education, Tianjin 300350, P. R. China.; ^6^ School of Energy and Power Engineering, Nanjing University of Aeronautics and Astronautics, Nanjing 210016, P. R. China.; ^7^ State Key Laboratory of Integrated Optoelectronics, Key Laboratory of Automobile Materials of MOE, and School of Materials Science and Engineering, Jilin University, Changchun, China.; ^8^ LONGi Central R&D Institute, LONGi Green Energy Technology Co. Ltd., Xi’an 710018, China.; ^9^ State Key Laboratory for Mechanical Behavior of Materials, Xi’an Jiaotong University, Xi’an, Shaanxi 710049, P. R. China.; ^10^ Laboratory of Photonics and Interfaces, Institute of Chemical Sciences and Engineering, École Polytechnique Fédérale de Lausanne (EPFL), 1015 Lausanne, Switzerland.; ^11^ Academy for Advanced Interdisciplinary Studies, Nankai University, Tianjin 300071, P. R. China.

## Abstract

Solar-driven electrochemical CO_2_ reduction to multicarbon (C_2+_) products presents a promising avenue for artificial photosynthesis, yet remains constrained by high overpotentials and limited conversion efficiency. Here, we developed an alkaline environment-modulated prereduction strategy that capitalizes on the divergent thermodynamic stabilities of precursors, enabling the precise synthesis of iodine-doped copper nanotopologies (Cu-I NTs). Featuring tunable under-coordination defects and stabilized high-energy adsorption sites, the Cu-I NTs achieve an onset potential of only −0.37 V versus the reversible hydrogen electrode for C_2+_ formation. Operando Raman measurement and density functional theory calculation reveal that the tailored surface topologies favor Cu-CO rotation adsorption, enabling dynamic reorientation from bridge- to atop-binding configurations, which collectively lowers the barrier for CO-CO coupling. Impressively, powering the electrolyzer by a perovskite/silicon tandem together with a single-junction silicon photovoltaic device, the system delivers a photocurrent of 12.34 mA cm^−2^ at 2.17 V under standard AM 1.5 illumination, achieving a solar-to-C_2+_ conversion efficiency of 8.64%.

## Teaser

Cu-I nanotopologies enable low-overpotential C_2+_ formation and 8.64% solar-to-C_2+_ efficiency.

## INTRODUCTION

Artificial photosynthesis offers a sustainable approach for converting CO_2_ into value-added fuels and chemicals by coupling efficient photovoltaic (PV) devices with electrocatalytic CO_2_ reduction (*1*, *2*). Among these systems, multicarbon (C_2+_) compounds, such as ethylene and ethanol, are particularly valuable due to their high energy density and utility as superior energy carriers (*3*).

To date, copper-based catalysts remain the only class of materials capable of catalyzing C_2+_ formation with appreciable efficiency (*4*). Accordingly, extensive efforts have focused on improving C_2+_ selectivity at high current densities (*5*–*7*). However, the large operating currents and associated overpotentials required by these catalysts are fundamentally mismatched with practical PV devices, severely limiting their deployment in integrated PV-EC artificial photosynthesis systems.

Recent strategies to lower the operating voltage of Cu catalysts, such as crystal-phase engineering (*8*, *9*) or noble-metal doping (*10*–*12*), have improved C_2+_ selectivity while lowering operating potentials, yet solar-to-C_2+_ conversion efficiencies in integrated PV-EC systems generally remain below 6.5%. These limitations are closely associated with the intrinsically demanding kinetics of C-C coupling (*13*). In addition, Cu-based catalysts often undergo dynamic structural evolution under reaction conditions, accompanied by continuous reconfiguration of local coordination environments, which adversely affects the production distribution of CO_2_ reduction.

In contrast to noble-metal modification and purely morphological tuning, topological structuring represents a distinct regulatory strategy for regulating atomic connectivity and local coordination environments, thereby influencing *CO adsorption and C-C coupling behavior (*14*–*16*). In this context, halide ions provide a unique handle for implementing such control. Owing to their strong coordination affinity with Cu and distinct thermodynamic properties, halides can dynamically interact with Cu species, influencing both electronic structure and reconstruction behavior (*17*–*19*). Notably, halide ions, particularly I^−^, can participate in redox-coupled interactions with Cu, actively driving structural evolution during electrochemical processes (*20*). Together with the markedly different solubility product constants of copper halides, these effects offer a mechanistic basis for programming catalyst reconstruction pathways, enabling direct control over the formation of Cu coordination environments under reaction conditions.

Here, we report an alkaline-triggered restructuring of a single CuI precursor with divergent thermodynamic stabilities in different alkaline electrolytes, enabling precise programming of Cu-I nanotopologies (NTs) to construct local coordination environments. As a result, thermodynamically stable regions preferentially reconstruct into Cu-I nanowires (NWs), whereas less stable regions evolve into Cu-I nanoparticles (NPs). This topological differentiation intrinsically programs intermediate Cu-Cu coordination environments within the NP domains, which selectively stabilize atop-bound *CO intermediates (*CO_top_), lower the C-C coupling barrier, and enable C_2+_ formation at reduced overpotentials. Consequently, the NP-structured Cu-I NTs exhibit a low onset potential of −0.37 V versus reverse hydrogen electrode (versus RHE) for C_2+_ products, delivering a Faradaic efficiency (FE) of 29.8% and reaching a peak C_2+_ FE of 78.7% at −0.61 V versus RHE. When integrated with low-cost perovskite PVs, the resulting system achieves a solar-to-C_2+_ conversion efficiency of 8.64%, a benchmark that rises to 9.15% under operation with III-V solar cells. Overall, this work highlights a reaction pathway engineered approach to coordination control through electrolyte-mediated reconstruction of CuI precursors, offering a mechanistically grounded strategy for efficient solar-driven CO_2_-to-C_2+_ conversion.

## RESULTS

### Synthesis and characterization of Cu-I NTs

**Fig. 1.**
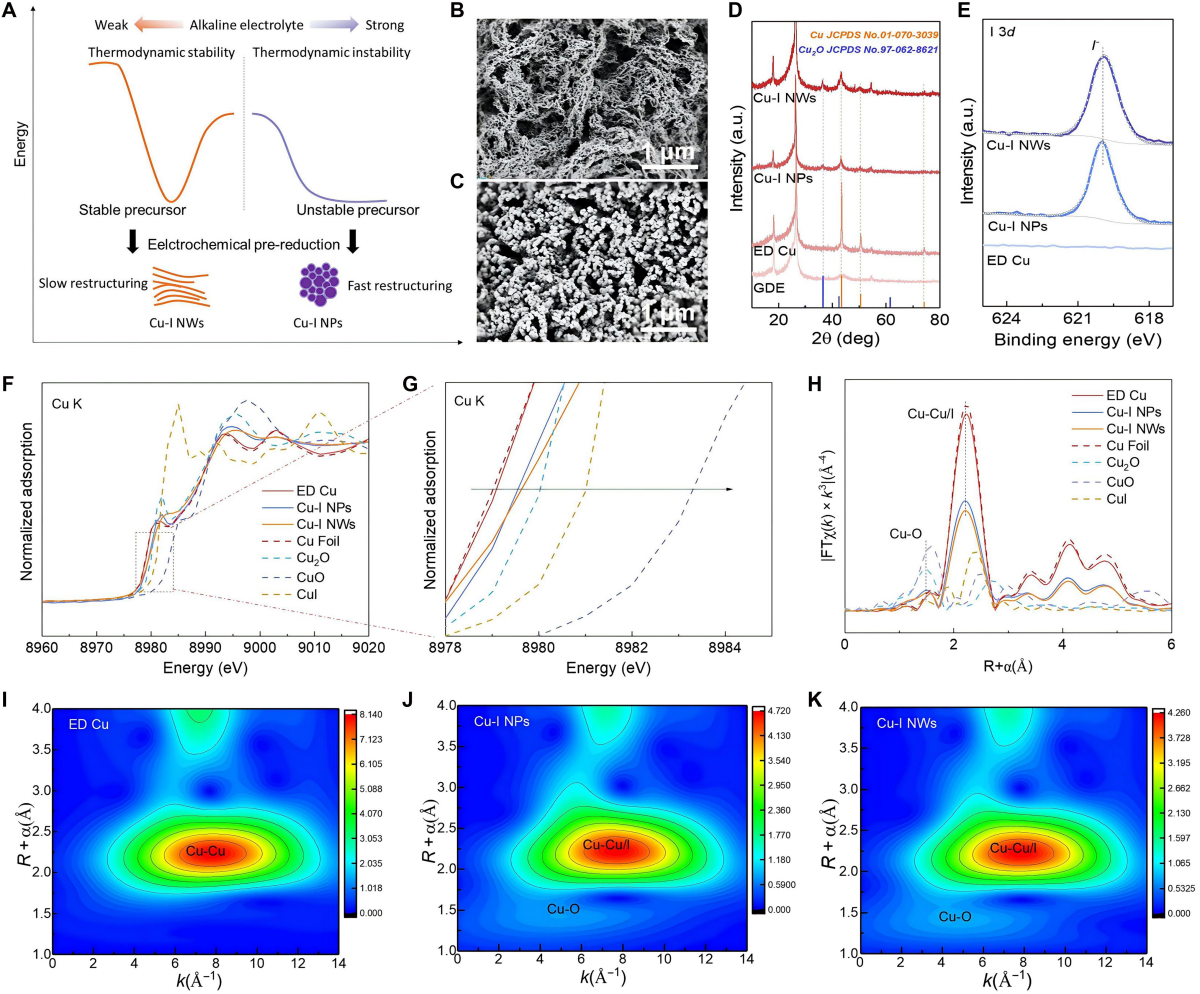
Synthesis and characterization of the catalysts. (**A**) Mechanistic schematic of Cu-I NTs: NWs and NPs. SEM images of (**B**) Cu-I NWs and (**C**) Cu-I NPs catalysts. (**D**) X-ray diffractograms of Cu-I NPs, Cu-I NWs, ED Cu, and GDE substrate. High-resolution XPS spectra of I 3*d* (**E**) of Cu-I NPs, Cu-I NWs, and ED Cu. (**F** and **G**) Cu K-edge XANES spectra. (**H**) Fourier-transformed EXAFS spectra. (**I** to **K**) Wavelet transform analysis of the EXAFS data for ED Cu, Cu-I NPs, and Cu-I NWs.

CuI precursor films were first synthesized via electrochemical treatment of electrodeposited Cu (ED Cu) in a KI electrolyte. Subsequently, these films underwent an alkaline environment-modulated prereduction step (Fig. 1A) by applying a current density of −30 mA cm^−2^ for 5 min. The formation of Cu-I NTs exhibited a strong dependence on OH^−^ concentration. Prereduction in weakly alkaline 1 M KHCO_3_ (pH 8.68) generated NWs (Cu-I NWs, Fig. 1B), whereas strongly alkaline 1 M KOH (pH 13.94) triggered the spontaneous assembly of NPs (Cu-I NPs) into mesoporous architectures (Fig. 1C). Note that these structures differed from both starting ED-Cu sample and CuI precursor (figs. S1 and S2). This alkaline-triggered restructuring behavior can be rationalized by the divergent thermodynamic stabilities of the CuI-derived intermediates under different pH environments, which are governed by their solubility products (Ksp) and coordination affinities (*21*). Under strongly alkaline conditions (1 M KOH), elevated OH^−^ concentrations drive the rapid conversion of CuI to CuOH, followed by immediate dehydration to Cu_2_O (figs. S3 to S5) (*22*). The substantially lower Ksp of Cu_2_O, together with the high OH^−^ concentration, promotes rapid iodide leaching, thereby enabling porous structure formation. In contrast, CuI remains comparatively stable under weakly alkaline conditions, including 1 M KHCO_3_, 0.1 M KHCO_3_ (pH 8.49), and 0.1 M KOH (13.01), leading to the formation of NW structures during the prereduction process (figs. S6 and S7).

X-ray diffraction (XRD) patterns confirmed the distinct phases of ED Cu and CuI (Fig. 1D and fig. S8). For both Cu-I NPs and Cu-I NWs, XRD and x-ray photoelectron spectroscopy (XPS) revealed coexisting Cu^0^ and Cu^+^ species, with Cu^+^ as the dominant state based on Cu LMM Auger spectra (Fig. 1, D and E, and fig. S9). XPS detected I^−^ species with an I 3*d* peak at 620.09 eV (Fig. 1E and fig. S8), though reduced intensity suggested partial iodine loss during reduction. High-resolution transmission electron microscopy (TEM) of Cu-I NPs showed lattice spacings of 0.209 and 0.180 nm for Cu(111) and Cu(100), respectively, while lattice spacings of 0.247 nm indicated the presence of surface Cu_2_O(111) planes (fig. S10). These ex situ valence-state analyses were not intended to identify the operando active state, as surface oxidation during sample transfer was unavoidable. Instead, these measurements served as complementary fingerprints reflecting the distinct precursor-derived structures before reaction.

Cu K-edge XAS showed that ED Cu closely resembled metallic Cu, indicating minimal valence deviation. In contrast, Cu-I NPs and NWs exhibited edge positions between Cu foil and Cu_2_O, suggesting mixed Cu^0^/Cu^+^ states (Fig. 1, F and G). Extended x-ray absorption fine structure (EXAFS) R-space fitting revealed dominant Cu-Cu coordination in ED Cu, while Cu-I samples showed mixed Cu-Cu/I coordination and minor Cu-O contributions (Fig. 1H). Coordination numbers were lower for Cu-I NPs (7.2) and NWs (5.6) than bulk Cu, consistent with E-space analysis (fig. S11 and table S1). Wavelet transform analyses further confirmed the coexistence of Cu-Cu and Cu-I scattering paths, together with minor Cu-O contributions (Fig. 1, I and K, and fig. S12), indicative of under-coordinated Cu architecture.

### Electrocatalytic CO_2_ reduction performance of Cu-I NPs

**Fig. 2.**
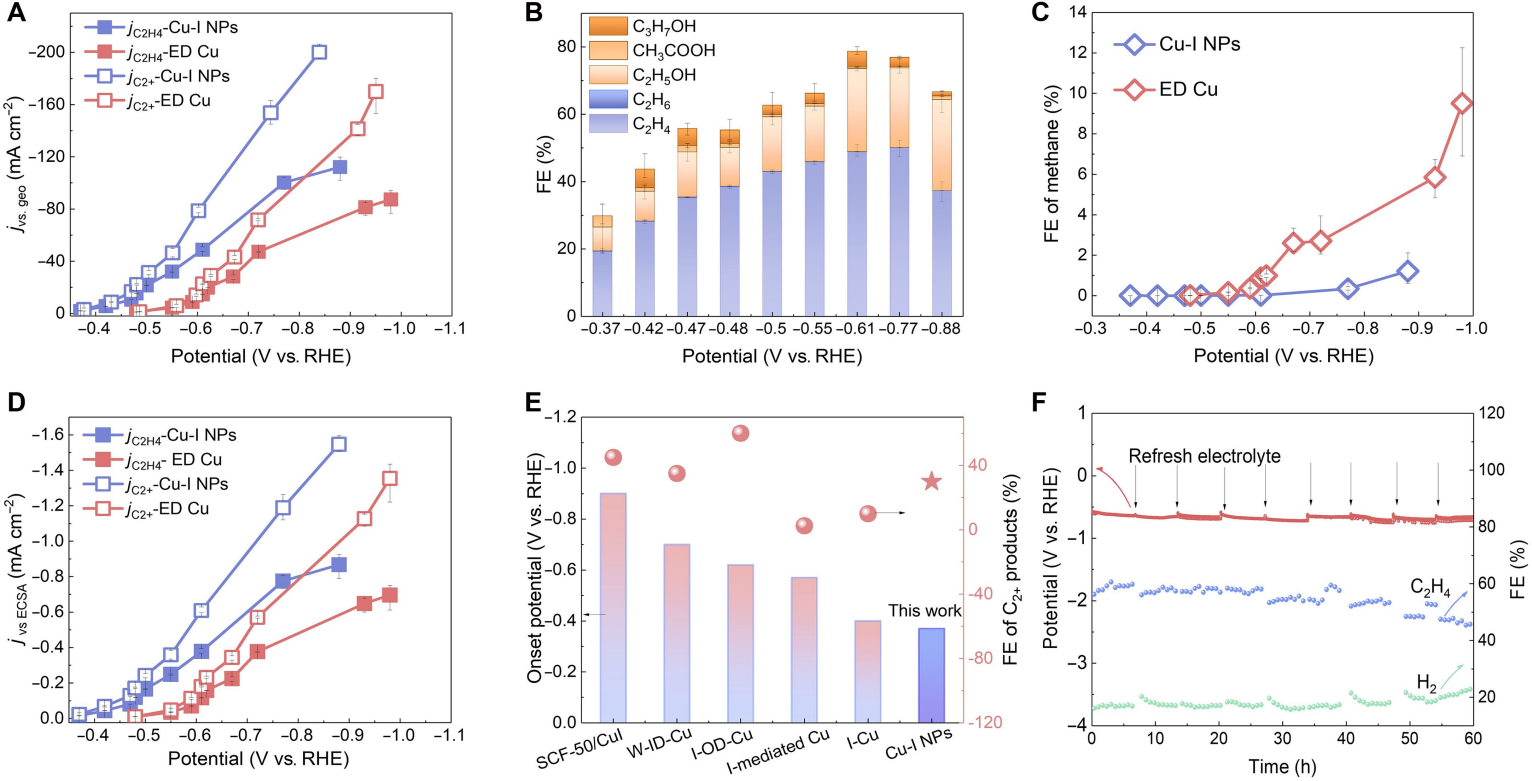
Electrochemical analysis of Cu-I NPs. (**A**) Partial current density *j*_vs.geo_ of C_2_H_4_ and C_2+_ products versus potential of Cu-I NPs and ED Cu catalyst. (**B**) FE of CO_2_ reduction to multicarbon products as a function of applied potential over the Cu-I NPs. (**C**) FE of CH_4_ over the Cu-I NPs and ED Cu catalyst as a function of applied potential. (**D**) Partial current density *j*_vs.ECSA_ of C_2_H_4_ and C_2+_ products versus potential of Cu-I NPs and ED Cu catalyst. *j*_vs.geo_ and *j*_vs.ECSA_ denote the current density normalized against the geometric area and active area of the catalysts. (**E**) Summary of iodine-modified Cu catalysts for FE toward multicarbon products and their onset potentials in flow cells. (**F**) FE and applied potential of C_2_H_4_ and H_2_ for 60 hours of electrolysis on Cu-I NPs at −100 mA cm^−2^.

Electrochemical CO_2_RR was evaluated in an alkaline flow cell. Under CO_2_ flowing condition, linear sweep voltammetry showed higher cathodic currents over Cu-I NPs electrode (fig. S13). ED Cu required high onset potentials of −0.48 V versus RHE and a peak FE of 73.3% at −0.93 V for C_2+_ products, while Cu-I NPs demonstrated markedly enhanced CO_2_ reduction performance, achieving C_2+_ formation at a much lower onset potential of −0.37 V and a higher peak FE of 78.7% at −0.61 V (Fig. 2, A and B; fig. S14; and tables S2 and S3). The remaining FE, completing the ∼100% total (*23*–*25*), arises mainly from hydrogen evolution and minor C_1_ products, with the full product distribution provided in tables S2 and S3. The ED Cu catalysts exhibited over 170-fold increase in the FE for unwanted methane formation at −100 mA cm^−2^, particularly at cathode potentials beyond −0.6 V versus RHE (Fig. 2C and fig. S15). This behavior is consistent with prior reports showing that, on highly coordinated metallic Cu surfaces, strongly negative potentials favored further hydrogenation of *CO to CH_4_ rather than C-C coupling (*26*, *27*). In contrast, the reduced methane selectivity on Cu-I NPs correlated with their lower average Cu-Cu coordination number (CN ≈ 7.2, Fig. 1H), which enhanced *CO stabilization and promotes C-C coupling pathways over *CO hydrogenation (*16*, *28*, *29*).

Moreover, the Cu-I NWs that were prereduced in KHCO_3_ solution, exhibited a decreased selectivity for multicarbon products despite their lower onset potentials (fig. S16). EXAFS analysis indicated that Cu-I NWs had an even lower coordination number (CN ≈ 5.7, Fig. 1H), which likely led to over-stabilization of *CO intermediates (*30*). Such excessively strong *CO binding had been reported to suppress *CO dimerization by favoring sequential hydrogenation pathways, thereby diminishing C_2+_ selectivity. These observations indicated that intermediate coordination environments, rather than maximally under-coordinated sites, are optimal for selective C-C coupling. Control experiments using Cu(OH)_2_ formed after prolonged alkaline treatment confirmed that this phase required substantially more negative potentials (−0.50 V versus RHE) than Cu-I NPs (−0.37 V versus RHE), indicating it was unlikely to be the intrinsically active species responsible for the enhanced C_2+_ performance (fig. S17).

Furthermore, specific current densities normalized to the electrochemically active surface area (ECSA) revealed that Cu-I NPs exhibited higher intrinsic activity for C_2_H_4_ dominated C_2+_ products than ED Cu catalysts, especially at cathode potentials more negative than −0.5 V versus RHE (Fig. 2D and fig. S18). These outstanding performances confirmed that the Cu-I NPs material exhibits the lowest onset potential among iodine-modified Cu-based catalysts for the electrosynthesis of C_2+_ products from CO_2_ (Fig. 2E and table S4) (*31*–*35*). While the I-OD-Cu catalyst delivered a 60% FE for C_2+_ products at −0.62 V versus RHE, our catalyst achieved nearly 80% at a slightly less negative potential (−0.61 V versus RHE), underscoring its enhanced performance and selectivity.

Notably, the Cu-I NPs-based system demonstrated stable operation at a current density of −100 mA cm^−2^ for 60 hours maintaining an average FE of 55% for C_2_H_4_ production throughout the test period (Fig. 2F). As the electrolysis proceeded, the C_2_H_4_ selectivity gradually decreased to ∼45%, accompanied by an increase in H_2_ to above 22%, indicating the onset of performance decay. This degradation was primarily attributed to carbonate accumulation on the backside of the gas diffusion electrode (GDE), which progressively shifts its wettability from hydrophobic to hydrophilic, thereby disrupting the triple-phase boundary, limiting CO_2_ transport, and promoting the hydrogen evolution reaction (*36*, *37*). Postreaction analyses confirmed that the catalyst structure remained intact with retained iodine species, suggesting that the decay mainly originated from electrode environment evolution rather than intrinsic catalyst instability (figs. S19 and S20). These findings highlighted carbonate management as a key factor for long-term operation, and point to acidic electrolytes as a promising strategy to suppress carbonate formation and improve stability.

Postreaction characterizations by scanning electron microscopy (SEM) and XRD showed that both Cu-I NPs and ED Cu largely preserved their overall surface morphology and crystalline framework after CO_2_ reduction (figs. S21 and S22), indicating robust structural stability under operating conditions. Notably, residual iodine signals were still detectable in the Cu-I NPs samples after electrolysis verified by XPS and inductively coupled plasma optical emission (fig. S23 and table S5). The observed 0.20 eV shift in the I 3*d* peak after electrolysis indicated increased electron density at iodine sites, reflecting changes in local coordination or defect formation during electrochemical restructuring (*38*, *39*). However, we emphasized that these postreaction, ex situ analyses did not imply continuous surface coverage or lattice incorporation of iodine under steady-state reaction conditions. Rather, the persistence of iodine after operation suggested incomplete leaching during electrochemical cycling, consistent with its strong affinity toward Cu species (*17*, *40*), and supported its role in modulating precursor reconstruction and coordination environments prior to and during catalyst activation.

### Mechanistic insights into Cu-I NPs for enhanced C_2+_ selectivity

**Fig. 3.**
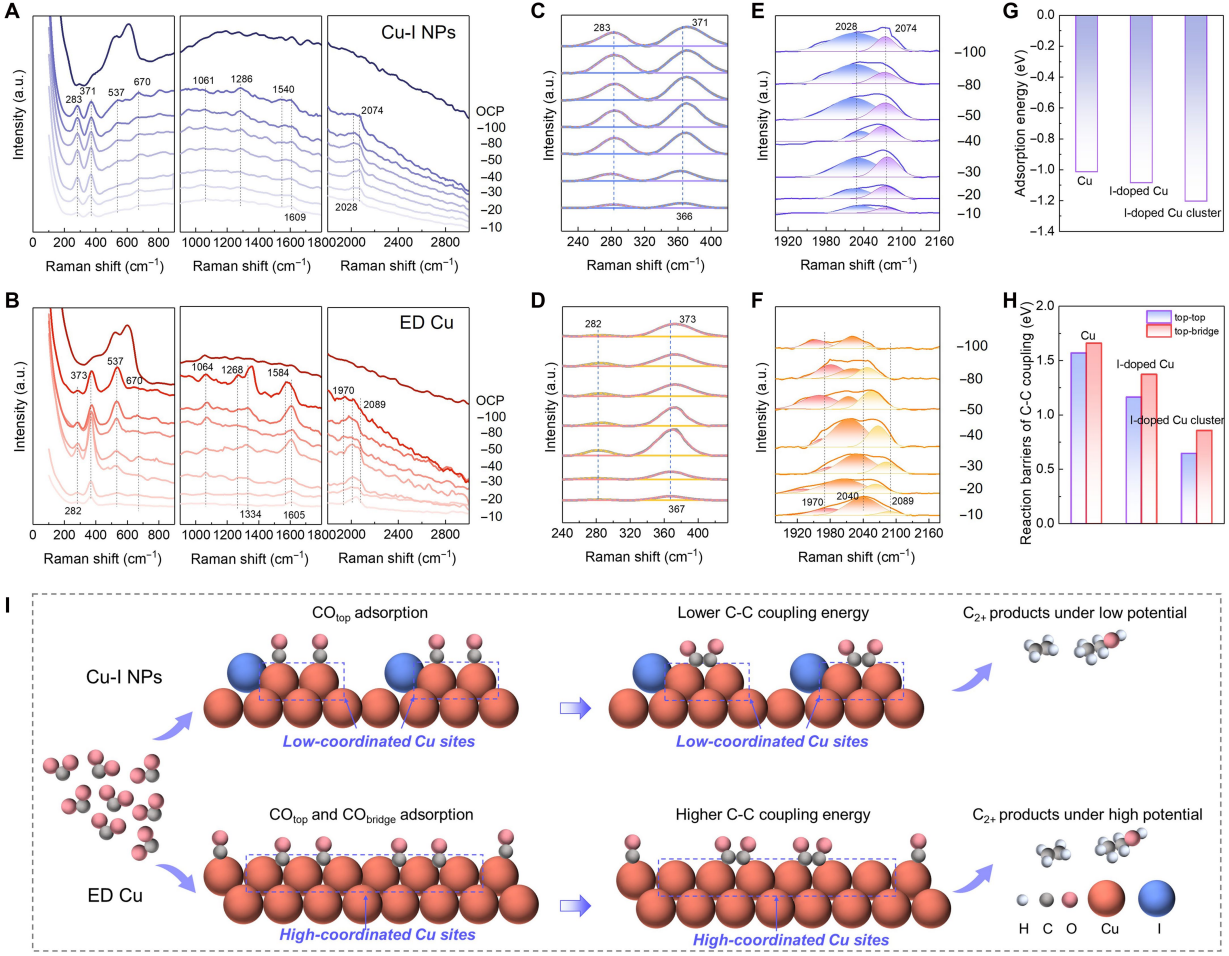
Reaction mechanism on Cu-I NPs catalysts. Operando Raman spectra of (**A**) Cu-I NPs and (**B**) ED Cu during electrochemical reduction of CO_2_ at various current densities (−10 to −100 mA cm^−2^) in 1.0 M KOH. Spectra were recorded in three distinct wave number regions: low–wave number range 100 to 900 cm^−1^, medium–wave number range 900 to 1800 cm^−1^, and high–wave number range 1800 to 3000 cm^−1^. The fitting results of operando Raman spectra in two distinct wave number regions: (**C** and **D**) wave number range 220 to 435 cm^−1^, (**E**) and (**F**) wave number range 1880 to 2180 cm^−1^. (**G**) CO_top_ adsorption energies on Cu (100) facets of Cu, I-doped Cu, and I-doped Cu cluster. (**H**) Reaction barriers of C-C coupling on Cu (100) facets of Cu, I-doped Cu, and I-doped Cu cluster. (**I**) Schematic illustration of the CO_2_ reduction reaction mechanism on Cu-I NPs and ED Cu catalysts.

Operando Raman spectroscopy revealed key vibrational bands on both Cu-I NPs and ED Cu catalysts during CO_2_ reduction at current densities from −10 to −100 mA cm^−2^ (Fig. 3, A to F), with all observed bands summarized in table S6 (*41*–*44*).

Typically, clear signatures of adsorbed *CO intermediates were observed, including Cu-C rotation modes at 281–283 cm^−1^, Cu─C stretching vibrations at 366–373 cm^−1^, and prominent C≡O stretching bands between 1920 to 2100 cm^−1^ (Figs. 3, C to F, and fig. S24). For the ED-Cu surface, the three deconvoluted bands at ∼1970, ∼2040, and ∼2089 cm^−1^ can be assigned to *CO_bridge_ and atop-bound *CO species (low-frequency and high-frequency bands), respectively, consistent with well-defined adsorption configurations on more uniform Cu sites (*45*).

Notably, the intensity of the 283 cm^−1^ peak was substantially higher on Cu-I NPs compared to ED Cu. The intensity ratio of the 281 to 283 cm^−1^ (P_1_) to 366 to 373 cm^−1^ (P_2_) peaks (P_1_/P_2_), which reflects the relative stabilization of the Cu-CO rotational adsorption configuration, was four and five times greater on Cu-I NPs under moderate current densities and further increased to a ninefold enhancement at −100 mA cm^−2^ (fig. S25). This pronounced enhancement indicated a higher population of dynamically adsorbed *CO species with increased configurational freedom, arising from under-coordinated Cu sites generated via alkaline-triggered surface restructuring. These under-coordinated sites provided sufficient stabilization of *CO while allowing the intermediates to access multiple adsorption geometries, including the kinetically favorable *CO_top_ configurations. Residual iodine species further modulated the local electronic environment of Cu, fine-tuning *CO binding strength and promoting this flexible adsorption behavior. Such sites favor *CO adsorption with increased configurational freedom, thereby facilitating the formation of *CO_top_ configurations that are kinetically favorable for C-C coupling (*16*, *46*–*48*). This adsorption behavior provides a direct spectroscopic basis for the reduced onset potential and enhanced C_2+_ selectivity observed for Cu-I NPs (Fig. 2, A and B). The apparent reduction in distinguishable *CO stretching features at higher current densities did not indicate fewer adsorption configurations, but rather the rapid consumption of reactive *CO intermediates during C-C coupling or CO hydrogenation process (*49*).

In contrast to ED Cu, which exhibited discernible Raman features assignable to *OH (537 cm^−1^) and transient carbon-oxygen intermediates in the 1334 to 1356 and 1540 to 1609 cm^−1^ regions (*50*), Cu-I NPs showed markedly weaker signals in these ranges under identical reaction conditions (table S6). Control experiments on bare carbon substrates under open-circuit conditions confirmed that these bands do not originate from the carbon paper (fig. S26). During CO_2_ reduction at cathodic potentials, the 1300 to 1500 cm^−1^ bands showed potential-dependent evolution, supporting their assignment to surface-generated oxygen-containing intermediates (e.g., carboxylate) rather than the substrate (*50*). The suppressed intensity of these features on Cu-I NPs reflected the more rapid conversion of early-stage CO_2_^−^ derived intermediates to *CO (*50*), rather than their absence, in a dynamic surface environment where *OH species did not accumulate to levels that perturb *CO adsorption. This behavior aligns with reports showing that moderate *OH coverage can support or even enhance C-C coupling, while excessive accumulation may lead to site blocking (*51*, *52*).

To further rationalize the origin of the distinct adsorption behavior, OH^−^ adsorption was used as an electrochemical probe to assess the surface facet distribution induced by catalyst restructuring, providing a structural basis for the subsequent density functional theory (DFT) modeling. Characteristic adsorption peaks at ∼0.37, ∼0.42, and ∼0.47 V versus RHE corresponded to Cu(100), Cu(110), and Cu(111), respectively (fig. S27) (*53*, *54*). By integrating the corresponding peak and analyzing the area ratios of each facet (table S7), it was found that Cu-I NPs exhibited a higher proportion of Cu(100) and Cu(110) facets compared to ED Cu, likely due to in situ surface reconstruction under prereduction. Given the known role of Cu(100) in facilitating C-C coupling (*55*), this facet enrichment correlates with the enhanced intrinsic activity of Cu-I NPs toward C_2+_ product formation.

DFT calculations were guided by EXAFS data showing a reduced average Cu-Cu coordination number of 7.2 ± 0.8 for Cu-I NPs (Fig. 1H and table S1). To systematically decouple the effects of under-coordinated sites and electronic factors in the physical samples, three distinct surface models were constructed: (i) pristine Cu, representing the pure ED Cu baseline; (ii) I-doped Cu, a flat surface model incorporating iodine to isolate its purely electronic effect without the influence of low-coordination Cu sites; and (iii) an I-doped Cu cluster. The cluster model, featuring active Cu atoms with a specifically reduced coordination number, serves as a direct theoretical proxy for the optimal Cu-I NPs catalyst, capturing both its highly under-coordinated sites and the electronic influence of residual iodine, with *CO adsorption modeled specifically at the atop site on an under-coordinated Cu atom (Fig. 3, G and H, and figs. S28 to S32) (*32*). Calculations show that *CO binding strength and C-C coupling kinetics improve from pristine Cu to I-doped Cu to the I-doped cluster, consistent with operando Raman evidence of enhanced *CO_top_ stabilization. Complementary experiments on Cu NPs without iodine (prepared from ED Cu in 0.1 M K_2_SO_4_; fig. S33) exhibit higher onset potentials and reduced C_2+_ selectivity, supporting the DFT-predicted role of iodine in promoting *CO stabilization and C-C coupling. The I-doped Cu cluster is a theoretical construct, not an isolable material, capturing the dominant local coordination motifs within Cu-I NPs. Collectively, these results demonstrate that iodine-modulated under-coordinated Cu sites stabilize *CO_top_, lower C-C coupling barriers, and enable high C_2+_ selectivity at reduced overpotentials (Fig. 3I).

### Unassisted solar-driven CO_2_ electroreduction to C_2+_ products using Cu-I NPs catalyst

**Fig. 4.**
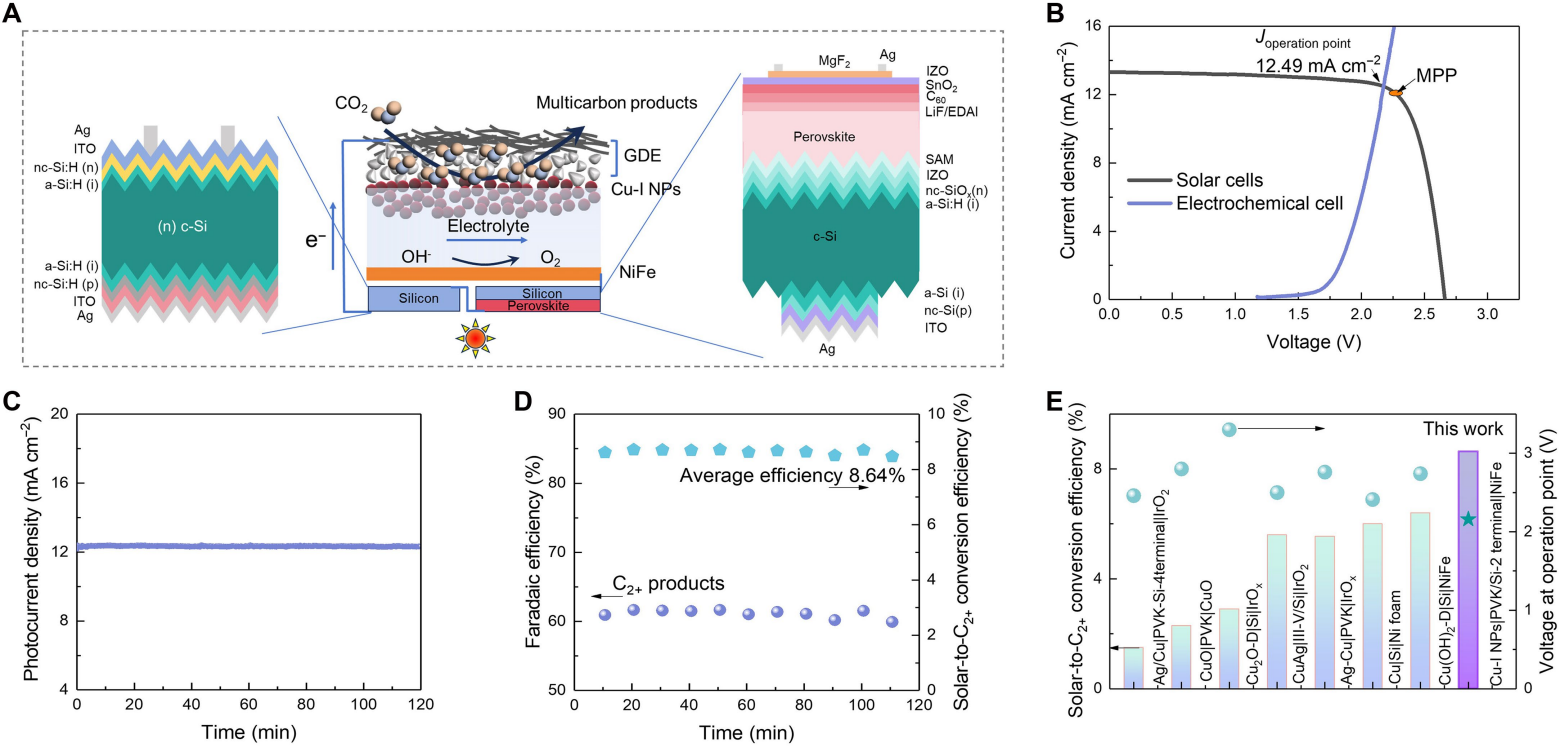
Photosynthesis of multicarbons from solar-driven CO_2_ reduction. (**A**) A solar-driven system for multicarbon compound synthesis using series-connected perovskite/silicon tandem and single-junction silicon solar cells. (**B**) Linear sweep voltammograms of the solar cells and electrolysis cell. (**C**) Current density of the unassisted PV-EC system under standard AM 1.5 G illumination. (**D**) FE toward multicarbons and the corresponding solar-to-multicarbons conversion efficiency of the unassisted PV-EC system under standard AM 1.5 G illumination. (**E**) Comparison of solar-to-C_2+_ efficiency and operating voltage with recent state-of-the-art PV-EC systems. For both (B) and (C), the electrolysis current density is normalized to the illuminated area of the solar cell (1.54 cm^2^). MPP denotes the maximum power point of the solar cell.

The two-electrode flow cell showed a remarkable activity, with a current density of −52 mA cm^−2^ achieved at a cell voltage of approximately 2.15 V, sustaining a FE of 41.37 and 60.07% for C_2_H_4_ and C_2+_ products formation, respectively (fig. S34). Given the voltage demands of the electrolyzer, we constructed a PV power supply composed of a perovskite/silicon tandem cell connected in series with a single-junction silicon solar cell (Fig. 4A). Under AM 1.5G illumination, this configuration provided an open-circuit voltage of 2.66 V and a short-circuit current density of 13.30 mA cm^−2^, resulting in a PCE of 27.40% (Fig. 4B). To ensure proper current matching with the PV output, the cathode area of the electrolyzer was optimized to 0.36 cm^2^. Accordingly, the measured electrolyzer current densities were normalized to the geometric area of the solar cell (1.54 cm^2^). On the basis of this normalization, the predicted operating point of the integrated device, defined by the intersection of the *J*-*V* curves of the solar cell and electrolyzer (Fig. 4B), was determined to be 12.49 mA cm^−2^ at an operating voltage of 2.17 V. This value closely aligned with the maximum power point (MPP) of the solar cell (12.12 mA cm^−2^ at 2.26 V), confirming efficient electrical coupling under operational conditions.

Unbiased operation under standard AM 1.5G illumination yielded an average FE of 61.13% for C_2+_ products generation at a steady current density of 12.34 mA cm^−2^ over 120 min (Figs. 4, C and D). When accounting for all C_2+_ gaseous and liquid products, it corresponded to a total solar-to-fuel efficiency of 8.64% (Fig. 4C and table S8). This benchmark performance, achieved by using earth-abundant photoabsorbers and catalysts, highlighted the scalability and sustainability of this approach. It also offered a competitive and cost-effective alternative to conventional photoelectrolysis systems (Fig. 4E and table S9) (*8*–*12*, *56*, *57*). The as-prepared NiFe LDH anode exhibited a nanosheet morphology with uniform Ni, Fe, and O distribution, and postreaction SEM/EDX confirmed that both the Cu-I NPs cathode and NiFe LDH anode maintained their structural integrity after PV-EC testing, demonstrating excellent electrode stability (figs. S35 to S37).

To demonstrate the generality of our PV-EC system, a high-efficiency III-V PV module was coupled to the Cu-I NPs catalyst, delivering 49.69 mA for 180 min with an average FE of 63.95% toward C_2+_ products and a solar-to-C_2+_ efficiency of 9.15% (fig. S38 and table S10). These results confirm that our catalyst integration strategy is effective across different PV technologies and can achieve higher conversion efficiencies with high-performance III-V solar cells.

## DISCUSSION

We have established an alkaline environment-modulated prereduction strategy to restructure CuI precursors into Cu-I NTs, achieving high activity for C_2+_ product formation at low potentials (−0.37 V versus RHE). The divergent thermodynamic stabilities of CuI under weak and strong alkaline conditions direct its evolution into either NWs or NPs, enabling topology-selective restructuring of the catalyst. This structural tuning of Cu-I NPs tailors under-coordinated sites, stabilizing the atop-bound *CO intermediate and lowering the barrier for C-C coupling, as confirmed by operando Raman spectroscopy and DFT calculations. We integrate the Cu-I NPs-based flow electrolyzer with a PV device comprising a perovskite/silicon tandem and single-junction silicon solar cells. This system achieves efficient solar-to-C_2+_ products conversion with efficiency of 8.64%, operating entirely without external bias. Collectively, our findings demonstrate a reaction pathway engineered approach to coordination control through electrolyte-modulated restructuring, offering a mechanistically informed route toward selective multicarbon electrosynthesis and integrated solar fuel production.

## MATERIALS AND METHODS

### Preparation of Cu substrate

An ultrathin Cu layer with a thickness of around 6 nm was first sputtered on the surface of GDE for increase the hydrophilicity and conductivity of the substrate, and then the GDE with ultrathin Cu as substrate for electrochemical deposit in the electrolyte including 49.8-g CuSO_4_·5H_2_O and 14.8-g C_6_H_5_Na_3_O_7_ with 945.6-ml H_2_O and 54.4-ml H_2_SO_4_ (98%) at −27.8 mA cm^−2^ for 100 s. The as-prepared GDE-supported Cu films were then rinsed thoroughly with deionized water and dried using compressed air.

### Preparation of CuI substrate

The Cu/GDE substrate underwent electrochemical roughening in 0.1 M KI solution through eight cycles of pulsed current treatment. Each cycle consisted of two sequential steps: (i) a chronoamperometric step at an applied current of 15 mA for 10 s; (ii) a chronoamperometric step at an applied current of 95 mA for 5 s. After treatment, the CuI films formed on the GDE were thoroughly rinsed with deionized water and dried using compressed air. These films were subsequently prereduced in a custom-built flow cell under a constant current density of −30 mA cm^−2^ for approximately 300 s in 1 M KOH.

### Preparation of NiFe-LDH catalysts

NiFe-LDH was prepared via a hydrothermal process following a previously reported procedure. In summary, 0.4 g of Fe(NO_3_)_3_·9H_2_O, 0.3 g of Ni(NO_3_)_2_·6H_2_O, and 0.3 g of urea (all from Aladdin) were dissolved in 80 ml of deionized water and transferred to a 100-ml Teflon-lined autoclave. A piece of Ni foam, pretreated by sequential cleaning with dilute HCl, ethanol, and deionized water, was placed upright along the inner wall of the vessel. The reaction was carried out at 120°C for 6 hours in a convection oven. Once cooled to room temperature, the resulting material was thoroughly rinsed with deionized water and dried using compressed air.

### Characterizations of catalysts

Surface morphology was examined using SEM (Apreo S LoVac). TEM images were obtained with a JEM-2800 instrument equipped with a field emission gun operating at 200 kV. Crystal structure analysis was performed via XRD (Rigaku) and Raman spectroscopy (Bruker Senterra II). Surface elemental composition was analyzed using XPS (Thermo Fisher Scientific ESCALAB 250Xi) with an Al Kα radiation source.

### Electrochemical measurements in flow cell

Catalytic activity was evaluated in a flow-type electrochemical cell using a Parstat 4000 potentiostat (Princeton Applied Research) with automated control. The electrochemical setup consisted of a three-electrode system, where Cu-based materials, Ni foam, and Ag/AgCl served as the working electrode, counter electrode, and reference electrode, respectively. All reported potentials were adjusted to account for 90% of the solution resistance (iR correction) and converted to the RHE scale using the following equation

$$V_{\text{RHE}}=V_{\text{Ag}/\text{AgCl}}+0.197+0.0591\;\text{pH}$$ (1)

CO_2_ electroreduction experiments were conducted using either a cation (Fumasep FKE-50, Fumatech) or an anion exchange membrane (Selemion) to isolate the cathodic and anodic chambers. Both compartments were supplied with 1 M KOH electrolyte, circulated at a flow rate of 5 ml min^−1^ via a peristaltic pump. CO_2_ gas was continuously fed into the cathode side at 23 ml min^−1^, controlled by a mass flow controller. Gaseous products were monitored in real time using an online gas chromatograph (FuLi GC9790Plus) equipped with a thermal conductivity detector (TCD) and dual flame ionization detectors (FIDs). H_2_ was quantified using a TCD, while CO and hydrocarbon products (CH_4_, C_2_H_4_, and C_2_H_6_) were analyzed with FID. Liquid-phase products were identified and quantified using ^1^H nuclear magnetic resonance (NMR) spectroscopy (Bruker, 400 MHz). For NMR measurements, 615 μl of the electrolyte was mixed with 5 μl of dimethyl sulfoxide (internal standard) and 80-μl D_2_O.

### In situ Raman spectroscopy

In situ Raman spectroscopy was conducted using a Horiba XPloRA confocal microscope (638-nm laser, charge-coupled device detector, and 600 grooves/mm grating). Measurements used a water immersion objective (Olympus 100x) protected by a Teflon film and a deionized water layer. CO_2_ electroreduction occurred in a custom spectro-electrochemical flow cell, using a Cu-based GDE working electrode, Pt mesh counter electrode, and KCl-saturated Ag/AgCl reference electrode. The cathodic compartment received aqueous 1 M KOH (2 ml min^−1^) and gaseous CO_2_ (20 ml min^−1^), separated from the anolyte by an AEM.

### XAS experiments

XAS experiments were conducted at beamline BL13SSW of the Shanghai Synchrotron Radiation Facility. Data reduction, data analysis, and EXAFS fitting were performed and analyzed with the Athena and Artemis programs of the Demeter data analysis packages (*58*) that use the FEFF6 program (*59*) to fit the EXAFS data.

### DFT calculations

First-principles calculations based on DFT were carried out using the plane-wave pseudopotential method with the Perdew, Burke, and Ernzerhof exchange-correlation functional, as implemented in the Vienna Ab initio Simulation Package (*60*, *61*). The electron-ion interactions were described using projected augmented wave pseudopotentials (*62*). DFT-D3(BJ) dispersion correction method was implemented to account for van der Waals interactions (*63*). The energy cutoff for plane-wave expansions was set to 400 eV, and the 3 × 3 × 1 Monkhorst-Pack grid k-points were used to sample the Brillouin zone integration. A 20-Å vacuum was applied to both the upper and lower surfaces of the model to eliminate image interactions. Structural optimizations were carried out until the maximum force on any atom fell below 0.02 eV/Å. Transition states were initially located through the climbing-image nudged elastic band method (*64*), followed by refinement using the dimer method with a convergence threshold of 0.05 eV/Å. During geometry optimization, the bottom three atomic layers were constrained to their bulk positions, while the upper one layer was allowed to fully relax. All calculations were performed for CO dimerization pathways across three distinct surface models.

### Solar-driven CO_2_ reduction

Solar cell performance was evaluated under standard AM 1.5G illumination using a xenon lamp solar simulator (Enli.Tec, Taiwan), the active PV cell area being 1.54 cm^2^ (correspond to the total active area of both tandem and single-junction cells). For solar-driven CO_2_ reduction, a two-electrode flow cell configuration was used in 1.5 M KOH, using NiFe-LDH as the anode and Cu-I NPs as the cathode catalysts. During operation, the solar cell was directly connected to the electrolyzer, and system performance was assessed under AM 1.5G light conditions. Chronoamperometric testing was conducted without external bias using a CHI equipment. Gaseous products were analyzed via online gas chromatography, while liquid-phase products in the electrolyte were quantified using ^1^H NMR spectroscopy, both during and after electrolysis.

## References

[1] V. Andrei, G. M. Ucoski, C. Pornrungroj, C. Uswachoke, Q. Wang, D. S. Achilleos, H. Kasap, K. P. Sokol, R. A. Jagt, H. Lu, T. Lawson, A. Wagner, S. D. Pike, D. S. Wright, R. L. Z. Hoye, J. L. MacManus-Driscoll, H. J. Joyce, R. H. Friend, E. Reisner, Floating perovskite-BiVO_4_ devices for scalable solar fuel production. Nature 608, 518–522 (2022). 35978127 10.1038/s41586-022-04978-6

[2] J. Liu, Y. He, L. Ding, H. Zhang, Q. Li, L. Jia, J. Yu, T. W. Lau, M. Li, Y. Qin, X. Gu, F. Zhang, Q. Li, Y. Yang, S. Zhao, X. Wu, J. Liu, T. Liu, Y. Gao, Y. Wang, X. Dong, H. Chen, P. Li, T. Zhou, M. Yang, X. Ru, F. Peng, S. i. Yin, M. Qu, D. Zhao, Z. Zhao, M. Li, P. Guo, H. Yan, C. Xiao, P. Xiao, J. Yin, X. Zhang, Z. Li, B. He, X. Xu, Perovskite/silicon tandem solar cells with bilayer interface passivation. Nature 635, 596–603 (2024). 39236747 10.1038/s41586-024-07997-7

[3] V. Andrei, I. Roh, J. Lin, J. Lee, Y. Shan, C. Lin, S. Shelton, E. Reisner, P. Yang, Perovskite-driven solar C_2_ hydrocarbon synthesis from CO_2_. Nat Catal. 8, 137–146 (2025).

[4] S. Nitopi, E. Bertheussen, S. B. Scott, X. Liu, A. K. Engstfeld, S. Horch, B. Seger, I. E. L. Stephens, K. Chan, C. Hahn, J. K. Nørskov, T. F. Jaramillo, I. Chorkendorff, Progress and perspectives of electrochemical CO_2_ reduction on copper in aqueous electrolyte. Chem. Rev. 119, 7610–7672 (2019). 31117420 10.1021/acs.chemrev.8b00705

[5] L. Chen, J. Chen, W. Fu, J. Chen, D. Wang, Y. Xiao, S. Xi, Y. Ji, L. Wang, Energy-efficient CO_(2)_ conversion to multicarbon products at high rates on CuGa bimetallic catalyst. Nat. Commun. 15, 7053 (2024). 39147764 10.1038/s41467-024-51466-8 PMC11327302

[6] M. Sun, J. Cheng, M. Yamauchi, Gas diffusion enhanced electrode with ultrathin superhydrophobic macropore structure for acidic CO_2_ electroreduction. Nat. Commun. 15, 491 (2024). 38225248 10.1038/s41467-024-44722-4 PMC10789815

[7] C. Zhu, Y. Song, X. Dong, G. Li, A. Chen, W. Chen, G. Wu, S. Li, W. Wei, Y. Sun, Ampere-level CO_2_ reduction to multicarbon products over a copper gas penetration electrode. Energy Environ. Sci. 15, 5391–5404 (2022).

[8] D. Zhong, Z. Zhao, Q. Zhao, D. Cheng, B. Liu, G. Zhang, W. Deng, H. Dong, L. Zhang, J. Li, J. Li, J. Gong, Coupling of Cu(100) and (110) facets promotes carbon dioxide conversion to hydrocarbons and alcohols. Angew. Chem. Int. Ed. Engl. 60, 4879–4885 (2021). 33231928 10.1002/anie.202015159

[9] G. Zhang, Z. Zhao, D. Cheng, H. Li, J. Yu, Q. Wang, H. Gao, J. Guo, H. Wang, G. A. Ozin, T. Wang, J. Gong, Efficient CO_2_ electroreduction on facet-selective copper films with high conversion rate. Nat. Commun. 12, 5745 (2021). 34593804 10.1038/s41467-021-26053-w PMC8484611

[10] J. Gao, H. Zhang, X. Guo, J. Luo, S. M. Zakeeruddin, D. Ren, M. Grätzel, Selective C–C coupling in carbon dioxide electroreduction via efficient spillover of intermediates as supported by operando Raman spectroscopy. J. Am. Chem. Soc. 141, 18704–18714 (2019). 31656072 10.1021/jacs.9b07415

[11] Gurudayal, J. W. Beeman, J. Bullock, H. Wang, J. Eichhorn, C. Towle, A. Javey, F. M. Toma, N. Mathews, J. W. Ager, Si photocathode with Ag-supported dendritic Cu catalyst for CO_2_ reduction. Energy Environ. Sci. 12, 1068–1077 (2019).

[12] G. Dayal, J. Bullock, D. F. Srankó, C. M. Towle, Y. Lum, M. Hettick, M. C. Scott, A. Javey, J. Ager, Efficient solar-driven electrochemical CO_2_ reduction to hydrocarbons and oxygenates. Energy Environ. Sci. 10, 2222–2230 (2017).

[13] Y. Yang, S. Louisia, S. Yu, J. Jin, I. Roh, C. Chen, M. V. F. Guzman, J. Feijóo, P. Chen, H. Wang, C. J. Pollock, X. Huang, Y. Shao, C. Wang, D. A. Muller, H. D. Abruña, P. Yang, Operando studies reveal active Cu nanograins for CO_2_ electroreduction. Nature 614, 262–269 (2023). 36755171 10.1038/s41586-022-05540-0

[14] N. Ye, K. Wang, Y. Tan, Z. Qian, H. Guo, C. Shang, Z. Lin, Q. Huang, Y. Liu, L. Li, Y. Gu, Y. Han, C. Zhou, M. Luo, S. Guo, Industrial-level CO_2_ to formate conversion on turing-structured electrocatalysts. Nat. Synth. 4, 799–807 (2025).

[15] W. Chen, M. Bao, F. Meng, B. Ma, L. Feng, X. Zhang, Z. Qiu, S. Gao, R. Zhong, S. Xi, X. Hai, J. Lu, R. Zou, Designer topological-single-atom catalysts with site-specific selectivity. Nat. Commun. 16, 574 (2025). 39794333 10.1038/s41467-025-55838-6 PMC11724105

[16] Y. Zhang, Y. Chen, X. Wang, Y. Feng, Z. Dai, M. Cheng, G. Zhang, Low-coordinated copper facilitates the *CH_2_CO affinity at enhanced rectifying interface of Cu/Cu_2_O for efficient CO_2_-to-multicarbon alcohols conversion. Nat. Commun. 15, 5172 (2024). 38890306 10.1038/s41467-024-49247-4 PMC11189494

[17] S. Ahrland, J. Chatt, N. R. Davies, The relative affinities of ligand atoms for acceptor molecules and ions. Q. Rev. Chem. Soc. 12, 265–276 (1958).

[18] Y. Kwon, Y. Lum, E. L. Clark, J. W. Ager, A. T. Bell, CO_2_ electroreduction with enhanced ethylene and ethanol selectivity by nanostructuring polycrystalline copper. ChemElectroChem 3, 1012–1019 (2016).

[19] T. Kim, G. T. R. Palmore, A scalable method for preparing Cu electrocatalysts that convert CO_2_ into C_2+_ products. Nat. Commun. 11, 3622 (2020). 32681030 10.1038/s41467-020-16998-9 PMC7368024

[20] E. Smith, D. Venkataraman, Deleterious effects of halides and solvents used in electronic device fabrication on the integrity of copper iodide thin-films. ChemPlusChem 87, e202200101 (2022). 35793411 10.1002/cplu.202200101

[21] J. G. Speight, *Lange’s Handbook of Chemistry, 16th edn*, (McGrawHill, New York, 2005).

[22] Y. Shang, D. Zhang, L. Guo, CuCl-intermediated construction of short-range-ordered Cu_2_O mesoporous spheres with excellent adsorption performance. J. Mater. Chem. 22, 856–861 (2012).

[23] Y. Hori, A. Murata, R. Takahashi, Formation of hydrocarbons in the electrochemical reduction of carbon dioxide at a copper electrode in aqueous solution. J. Chem. Soc. Faraday Trans. I 85, 2309–2326 (1989).

[24] Y. Hori, H. Ito, K. Okano, K. Nagasu, S. Sato, Silver-coated ion exchange membrane electrode applied to electrochemical reduction of carbon dioxide. Electrochim. Acta 48, 2651–2657 (2003).

[25] Y. Hori, I. Takahashi, O. Koga, N. Hoshi, Electrochemical reduction of carbon dioxide at various series of copper single crystal electrodes. J. Mol. Catal. A Chem. 199, 39–47 (2003).

[26] D. Ren, J. Fong, B. S. Yeo, The effects of currents and potentials on the selectivities of copper toward carbon dioxide electroreduction. Nat. Commun. 9, 925 (2018). 29500358 10.1038/s41467-018-03286-w PMC5834446

[27] K. P. Kuhl, E. R. Cave, D. N. Abramc, T. F. Jaramillo, New insights into the electrochemical reduction of carbon dioxide on metallic copper surfaces. Energy Environ. Sci. 5, 7050–7059 (2012).

[28] X. K. Lu, B. Lu, H. Li, K. Lim, L. C. Seitz, Stabilization of undercoordinated Cu sites in strontium copper oxides for enhanced formation of C_2+_ products in electrochemical CO_2_ reduction. ACS Catal. 12, 6663–6671 (2022).

[29] J. Zhu, J. Chen, X. Qi, J. Zhao, X. Jiang, W. Li, J. Liu, Electron-rich subnanometer Cu clusters facilitate CO–CO coupling in CO_2_ electroreduction. J. Am. Chem. Soc. 148, 4008–4019 (2026). 41570330 10.1021/jacs.5c12495

[30] J. Jiao, X. Kang, J. Yang, S. Jia, Y. Peng, S. Liu, C. Chen, X. Xing, M. He, H. Wu, B. Han, Steering the reaction pathway of CO_2_ electroreduction by tuning the coordination number of copper catalysts. J. Am. Chem. Soc. 146, 15917–15925 (2024). 38805725 10.1021/jacs.4c02607

[31] W. Ma, S. Xie, T. Liu, Q. Fan, J. Ye, F. Sun, Z. Jiang, Q. Zhang, J. Cheng, Y. Wang, Electrocatalytic reduction of CO_2_ to ethylene and ethanol through hydrogen-assisted C-C coupling over fluorine-modified copper. Nat Catal. 3, 478–487 (2020).

[32] M. Wu, R. Yang, J. Duan, S. Zhu, B. Chen, Z. Shi, Y. Liu, H. Li, B. Xia, T. Zhai, Polymer-halogen pockets steering *CO adsorption configurations for highly selective CO_2_ electroreduction. Adv. Mater. 37, e2504292 (2025). 40200674 10.1002/adma.202504292

[33] Y. Shi, Y. Wang, C. Dong, T. T. T. Nga, D. Wei, J. Wang, X. Zhao, M. Wang, K. Zhang, M. Li, F. Dong, S. Shen, Localized geometry determined selectivity of iodide-derived copper for electrochemical CO_2_ reduction. Adv. Energy Mater. 13, 2203896 (2023).

[34] X. Lv, Y. Yang, J. Lv, L. Ji, J. Wang, X. Wu, Z. Li, X. Li, Q. Liu, Z. Qi, Q. Lin, A. Wu, H. Wu, Iodine-mediated C-C coupling in neutral flow cell for electrochemical CO_2_ reduction. Adv. Funct. Mater. 34, 2311236 (2024).

[35] Z. Yan, M. Liu, Z. Guo, Q. Chen, Z. Xi, X. Sun, J. Yu, T. Wu, Trace iodine modified copper catalyst drives asymmetric C–C coupling in stable CO_2_ electroreduction. Adv. Funct. Mater. 35, 2420493 (2025).

[36] X. She, L. Zhai, Y. Wang, P. Xiong, M. M. J. Li, T. S. Wu, M. C. Wong, X. Guo, Z. Xu, H. Li, H. Xu, Y. Zhu, S. C. E. Tsang, S. P. Lau, Pure-water-fed, electrocatalytic CO_2_ reduction to ethylene beyond 1,000 h stability at 10 A. Nat. Energy 9, 81–91 (2024).

[37] B. Endrődi, A. Samu, E. Kecsenovity, T. Halmágyi, D. Sebők, C. Janáky, Operando cathode activation with alkali metal cations for high current density operation of water-fed zero-gap carbon dioxide electrolysers. Nat. Energy 6, 439–448 (2021). 33898057 10.1038/s41560-021-00813-w PMC7610664

[38] C. C. Perry, N. S. Faradzhev, T. E. Madey, D. H. Fairbrother, Electron stimulated reactions of methyl iodide coadsorbed with amorphous solid water. J. Chem. Phys. 126, 204701 (2007). 17552783 10.1063/1.2722749

[39] A. Liu, H. Zhu, W. Park, S. Kang, Y. Xu, M. Kim, Y. Noh, Room-temperature solution-synthesized p-type copper(I) iodide semiconductors for transparent thin-film transistors and complementary electronics. Adv. Mater. 30, e1802379 (2018). 10.1002/adma.201802379 29974529

[40] S. Ahrland, B. Tagesson, D. Tuhtar, M. Pouchard, P. Hagenmuller, A. F. Andresen, Thermodynamics of metal complex formation in aqueous solution. XIII. Enthalpy measurements on copper(I) and silver(I) halide systems. Acta Chem. Scand. 31, 625–634 (1977).

[41] A. M. Asiri, J. Gao, S. B. Khan, K. A. Alamry, H. M. Marwani, M. S. J. Khan, W. A. Adeosun, S. M. Zakeeruddin, D. Ren, M. Grätzel, Revisiting the impact of morphology and oxidation state of Cu on CO_2_ reduction using electrochemical flow cell. J. Phys. Chem. Lett. 13, 345–351 (2022). 34982561 10.1021/acs.jpclett.1c03957

[42] H. Li, P. Wei, D. Gao, G. Wang, In situ Raman spectroscopy studies for electrochemical CO_2_ reduction over Cu catalysts. Curr. Opin. Green Sustain. Chem 34, 100589 (2022).

[43] F. Shao, J. K. Wong, Q. H. Low, J. Lan, In situ spectroelectrochemical probing of CO redox landscape on copper single-crystal surfaces. Proc. Natl. Acad. Sci. U.S.A. 119, e2118166119 (2022). 35858341 10.1073/pnas.2118166119 PMC9304001

[44] D. Bohra, I. Ledezma-Yanez, G. Li, W. Jong, E. A. Pidko, W. A. Smith, Lateral adsorbate interactions inhibit HCOO^−^ while promoting CO selectivity for CO_2_ electrocatalysis on silver. Angew. Chem. Int. Ed. Engl. 131, 1359–1363 (2019). 10.1002/anie.201811667 PMC6391976 30444950

[45] W. Niu, J. Feng, J. Chen, L. Deng, W. Guo, H. Li, L. Zhang, Y. Li, B. Zhang, High-efficiency C_3_ electrosynthesis on a lattice-strain-stabilized nitrogen-doped Cu surface. Nat. Commun. 15, 7070 (2024). 39152122 10.1038/s41467-024-51478-4 PMC11329774

[46] X. Kong, J. Zhao, J. Ke, C. Wang, S. Li, R. Si, B. Liu, J. Zeng, Z. Geng, Understanding the effect of *CO coverage on C-C coupling toward CO_2_ electroreduction. Nano Lett. 22, 3801–3808 (2022). 35467883 10.1021/acs.nanolett.2c00945

[47] F. A. Rollier, V. Muravev, A. Parastaev, R. C. J. Poll, J. M. J. J. Heinrichs, B. Ligt, J. F. M. Simons, M. C. Figueiredo, E. J. M. Hensen, Restructuring of Cu-based catalysts during CO electroreduction: Evidence for the dominant role of surface defects on the C_2+_ product selectivity. ACS Catal. 14, 13246–13259 (2024). 39263539 10.1021/acscatal.4c02718 PMC11385435

[48] Y. Huang, A. D. Handoko, P. Hirunsit, B. S. Yeo, Electrochemical reduction of CO_2_ using copper single-crystal surfaces: Effects of CO* coverage on the selective formation of ethylene. ACS Catal. 7, 1749–1756 (2017).

[49] J. Meng, J. Freeze, L. Nowack, C. Li, H. Wang, H. D. Abruña, A. P. Willard, V. S. Batista, T. Lian, Competitive carbonate binding hinders electrochemical CO_2_ reduction to CO on Cu surfaces at low overpotentials. J. Am. Chem. Soc. 147, 25361–25371 (2025). 40637341 10.1021/jacs.5c04518 PMC12291447

[50] C. Long, X. Liu, K. Wan, Y. Jiang, P. An, C. Yang, G. Wu, W. Wang, J. Guo, L. Li, K. Pang, Q. Li, C. Cui, S. Liu, T. Tan, Z. Tang, Regulating reconstruction of oxide-derived Cu for electrochemical CO_2_ reduction toward n-propanol. Sci. Adv. 9, eadi6119 (2023). 37889974 10.1126/sciadv.adi6119 PMC10610896

[51] A. Herzog, M. L. Luna, H. S. Jeon, C. Rettenmaier, P. Grosse, A. Bergmann, B. R. Cuenya, Operando Raman spectroscopy uncovers hydroxide and CO species enhance ethanol selectivity during pulsed CO_2_ electroreduction. Nat. Commun. 15, 3986 (2024). 38734726 10.1038/s41467-024-48052-3 PMC11088695

[52] D. Zhong, Q. Fang, R. Du, Y. Jin, C. Peng, D. Cheng, T. Li, T. Zhao, S. Zhang, Y. Zheng, Q. Zhao, Y. Sun, J. Li, Selective electrochemical CO_2_ reduction to ethylene or ethanol via tuning *OH adsorption. Angew. Chem. Int. Ed. Engl. 64, e202501773 (2025). 39953915 10.1002/anie.202501773

[53] J. Gao, A. Bahmanpour, O. Kröcher, S. M. Zakeeruddin, D. Ren, M. Grätzel, Electrochemical synthesis of propylene from carbon dioxide on copper nanocrystals. Nat. Chem. 15, 705–713 (2023). 37024716 10.1038/s41557-023-01163-8 PMC10159857

[54] A. Tiwari, H. H. Heenen, A. S. Bjørnlund, T. Maagaard, E. Cho, I. Chorkendorff, H. H. Kristoffersen, K. Chan, S. Horch, Fingerprint voltammograms of copper single crystals under alkaline conditions: A fundamental mechanistic analysis. J. Phys. Chem. Lett. 11, 1450–1455 (2020). 32022563 10.1021/acs.jpclett.9b03728

[55] F. Calle-Vallejo, M. T. M. Koper, Theoretical considerations on the electroreduction of CO to C_2_ species on Cu(100) electrodes. Angew. Chem. Int. Ed. Engl. 52, 7282–7285 (2013). 23733719 10.1002/anie.201301470

[56] D. Ren, N. W. H. Loo, L. Gong, B. S. Yeo, Continuous production of ethylene from carbon dioxide and water using intermittent sunlight. ACS Sustain. Chem. Eng. 5, 9191–9199 (2017).

[57] T. N. Huan, D. A. D. Corte, S. Lamaison, D. Karapinar, L. Lutz, N. Menguy, M. Foldyna, S.-H. Turren-Cruz, A. Hagfeldt, F. Bella, M. Fontecave, V. Mougel, Low-cost high-efficiency system for solar-driven conversion of CO_2_ to hydrocarbons. Proc. Natl. Acad. Sci. U.S.A. 16, 9735–9740 (2019). 10.1073/pnas.1815412116 PMC6525546 30918130

[58] B. Ravel, M. Newville, Athena, Artemis, Hephaestus: Data analysis for X-ray absorption spectroscopy using IFEFFIT. J. Synchrotron Radiat. 12, 537–541 (2005). 15968136 10.1107/S0909049505012719

[59] S. I. Zabinsky, J. J. Rehr, A. Ankudinov, R. C. Albers, M. J. Eller, Multiple-scattering calculations of X-ray-absorption spectra. Phys. Rev. B 52, 2995–3009 (1995). 10.1103/physrevb.52.2995 9981373

[60] G. Kresse, J. Furthmüller, Efficiency of ab-initio total energy calculations for metals and semiconductors using a plane-wave basis set. Comput. Mater. Sci. 6, 15–50 (1996).

[61] G. Kresse, J. Furthmüller, Efficient iterative schemes for ab initio total-energy calculations using a plane-wave basis set. Phys. Rev. B. 54, 11169–11186 (1996). 10.1103/physrevb.54.11169 9984901

[62] G. Kresse, D. Joubert, From ultrasoft pseudopotentials to the projector augmented-wave method. Phys. Rev. B. 59, 1758–1775 (1999).

[63] S. Grimme, J. Antony, S. Ehrlich, H. Krieg, A consistent and accurate ab initio parametrization of density functional dispersion correction (DFT-D) for the 94 elements H-Pu. J. Chem. Phys. 132, 154104 (2010). 20423165 10.1063/1.3382344

[64] G. Henkelman, B. P. Uberuaga, H. Jónsson, A climbing image nudged elastic band method for finding saddle points and minimum energy paths. J. Chem. Phys. 113, 9901–9904 (2000).

