# Supplementary material for: Thermodynamic control of Cu-I nanotopologies enables solar-driven CO2-to-multicarbon conversion
Source: Sci Adv. 2026 Jul 17;12(29):eaec6434. doi: 10.1126/sciadv.aec6434 (PMC13378565; doi:10.1126/sciadv.aec6434)
Supplement: Supplementary file 1 — Supplementary Text Figs. S1 to S38 Tables S1 to S10 [file sciadv.aec6434_sm.pdf]

Science Advances
AAAS

Supplementary Materials for

Thermodynamic control of Cu-I nanotopologies enables solar-driven CO_2-to-multicarbon conversion

Qixing Zhang *et al.*

Corresponding author: Bo He, hebo3@longi.com; Lijun Zhang, lijun_zhang@jlu.edu.cn; Michael Grätzel, michael.graetzel@epfl.ch; Xiaodan Zhang, xdzhang@nankai.edu.cn

Sci. Adv. **12**, eaec6434 (2026)
DOI: 10.1126/sciadv.aec6434

This PDF file includes:

Supplementary Text
Figs. S1 to S38
Tables S1 to S10

Supplementary Text

Calculation of solar-to-fuel (STF) conversion efficiency

The STF conversion efficiency for a PV-EC CO_2 reduction system, can be calculated as follows:

$$\eta_{STF} = \frac{j_{op} \times E_{fuel} \times FE_{fuel}}{P_{input}} \quad \text{(S1)}$$

Where j_{op} is the operating current density normalized to the working area of solar cell, P_{input} is the incident illumination power (100 mA cm^{-2}), E_{fuel} is the thermodynamic potential of the overall reaction for target product, FE_{fuel} is the Faradaic efficiency of target product.

Calculation of the Faradaic efficiency of CO_2 Reduction

The partial current density of the product *y* was calculated by the equation:

$$j_y = j_{total} \times FE(y) \quad \text{(S2)}$$

The Faradaic efficiency of the product *T* was calculated by the equation:

$$FE(y) = \frac{\text{Number of electrons required to produce } y}{\text{Total number of electrons for } CO_2 \text{ reduction}} \times 100\% \quad \text{(S3)}$$

Faradaic efficiency of gas products y:

$$N_{total} = \frac{I_0 \times t}{e} = \frac{I_0 \times t}{1.602 \times 10^{-19} \mathrm{C}/e} \quad \text{(S4)}$$

Where I_0 is the average current obtained from applied current. The time t is takes to fill the sample loop is: $t = \frac{V_0}{v}$. V_0 is the volume of the sample loop for hydrocarbons in our gas chromatograph. v is the flow rate of the CO_2 gas. The final equation of N_{total} is:

$$N_{total} = \frac{I_0 \times V_0}{e \times v} = \frac{I_0 \times V_0}{v \times 1.602 \times 10^{-19} \mathrm{C}/e} \quad \text{(S5)}$$

The equation of the number of electrons required to produce f (N_y) is:

$$N_{у} = x_0 \times n \times N_A \times m_1 e \qquad \text{(S6)}$$

Where x_0 is the achieved ppm of the *у*. m_1 is the number of electrons required to from 1 molecule of *у*.

According to the ideal gas law, n is the amount of gas in each sample loop V_0 under ambient temperature:

$$n = \frac{P \times V_0}{R \times T} \qquad \text{(S7)}$$

The final equation of $N_{у}$ is:

$$N_{у} = \frac{x_0 \times N_A \times m_1 e \times P \times V_0}{R \times T} \qquad \text{(S8)}$$

The equation of the Faradaic efficiency of the product *у* is:

$$\mathrm{FE}(у) = \frac{N_{у}}{N_{total}} = \frac{x_0 \times N_A \times m \times P \times v \times 1.602 \times 10^{-19}}{I_0 \times R \times T} \times 100\% \qquad \text{(S9)}$$

Faradaic efficiency of liquid products Л:

$$\mathrm{FE}(Л) = \frac{N_{Л}}{N_{total}} \qquad \text{(S10)}$$

The equation of the N_{total} is:

$$N_{total} = \frac{Q_0}{e} = \frac{Q_0}{1.602 \times 10^{-19} \mathrm{C}/e} \qquad \text{(S11)}$$

Where Q_0 is the total charge during the CO_2 reduction process.

The equation of the $N_{Л}$ is:

$$N_{Л} = C_{Л} \times V \times N_A \times m_2 e \qquad \text{(S12)}$$

Where V is the volume of catholyte. m_2 is the number of electrons required to from 1 molecule of ***Л***. $C_{Л}$ is the concentration of ***Л*** in the catholyte.

The equation of the Faradaic efficiency of the product ***Л*** is:

$$\mathrm{FE}(Л) = \frac{C_{Л} \times V \times N_A \times m_2 \times 1.602 \times 10^{-19}}{Q_0} \times 100\% \qquad \text{(S13)}$$

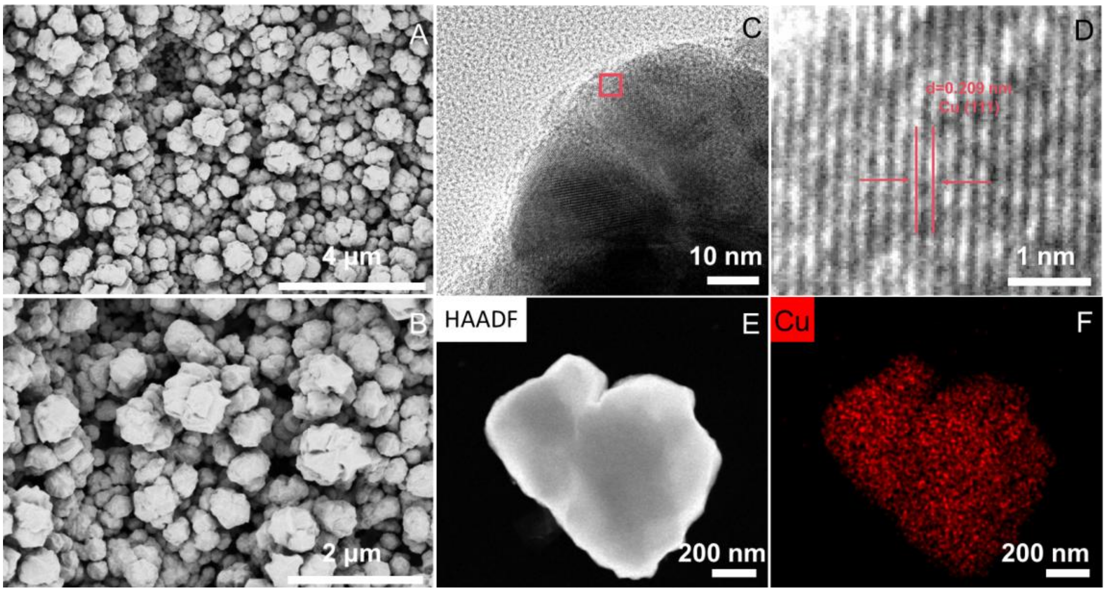

Figure S1. Morphological and compositional characterization of ED Cu samples. SEM images of ED Cu samples (A) and (B), (C) and (D) representative HRTEM images of ED Cu samples, (E) and (F) representative HAADF images and EDX element mappings of ED Cu samples.

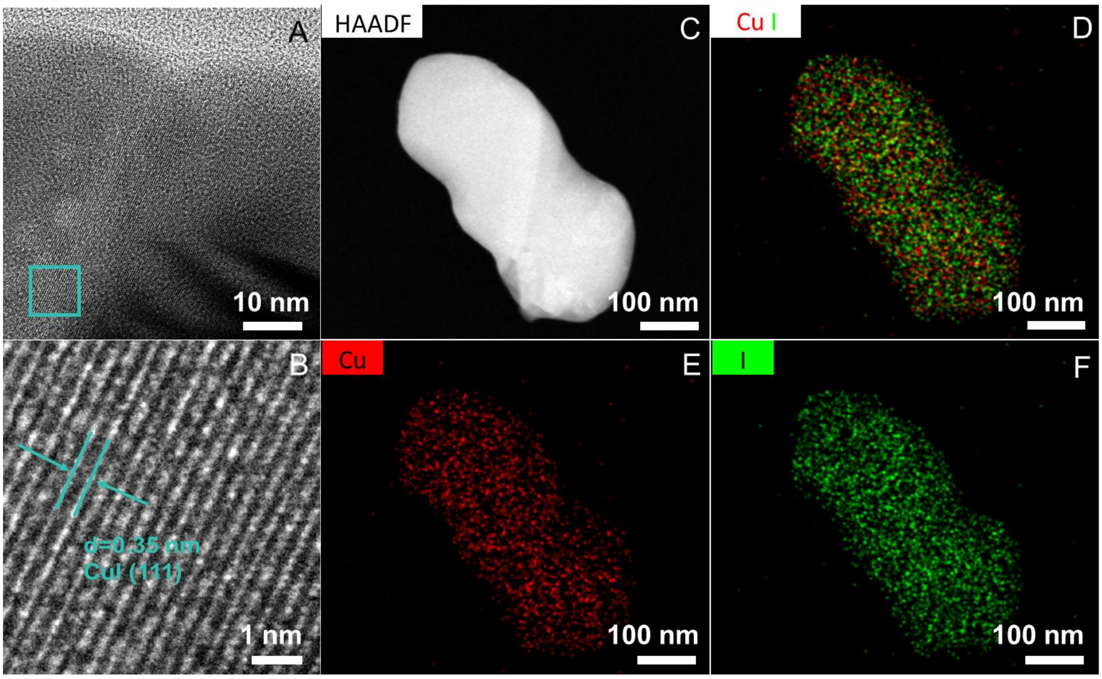

Figure S2. Morphological and compositional characterization of CuI samples. (A) and (B) representative HRTEM images of CuI samples, (C-F) representative HAADF images and EDX element mappings of CuI samples.

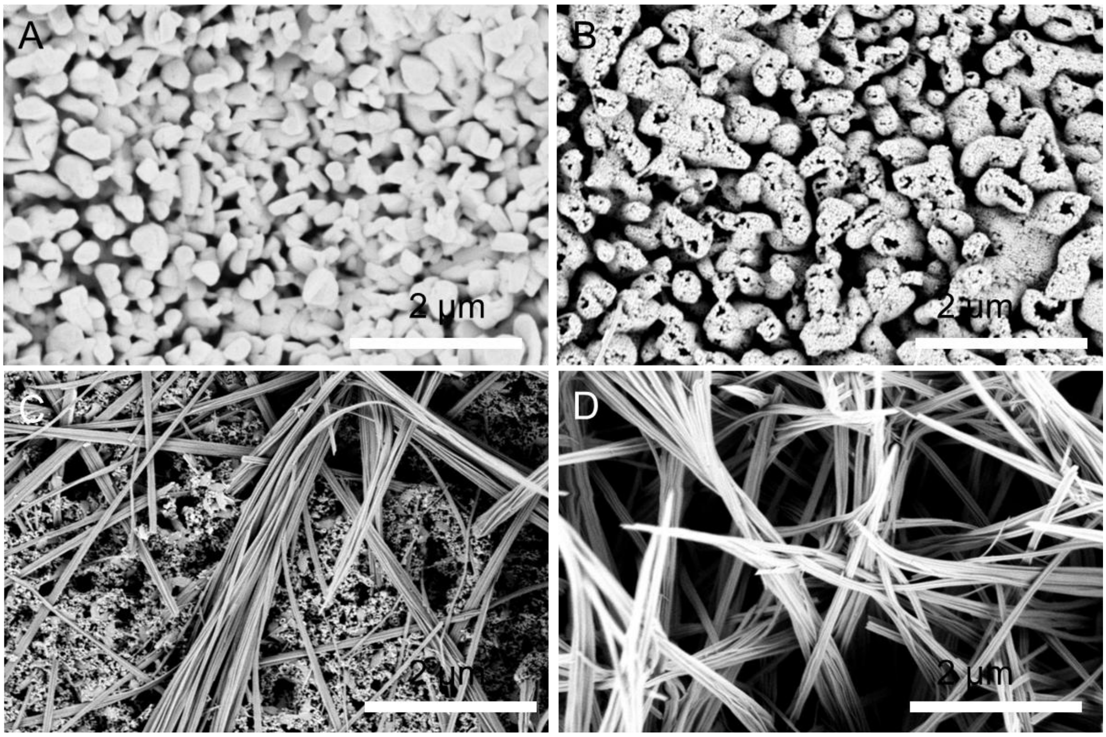

Figure S3. Time-dependent morphological evolution of CuI in alkaline electrolyte. Scanning electron microscopy (SEM) images of CuI samples immersed in 1 M KOH for (A) 0 s, (B) 10 s, (C) 600 s, and (D) 1200 s.

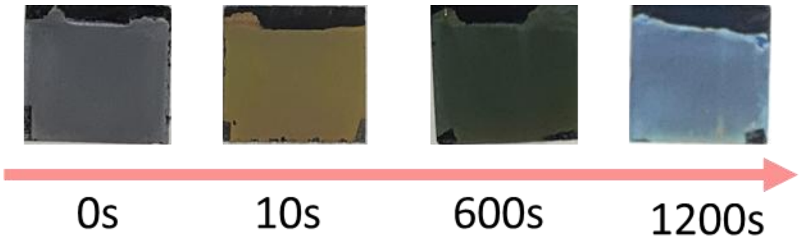

Figure S4. Macroscopic appearance evolution of CuI in alkaline solution. Digital photographs of CuI samples immersed in 1 M KOH for different durations.

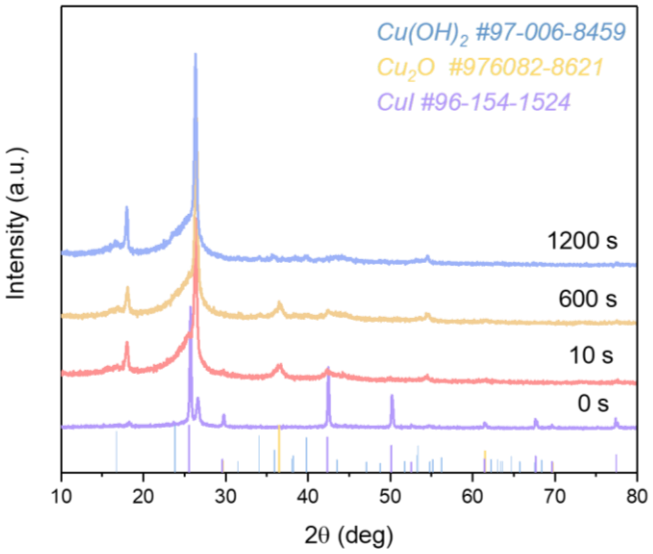

Figure S5. Phase evolution of CuI during immersion in alkaline electrolyte. X-ray diffraction of CuI samples immersed in 1 M KOH for different durations.

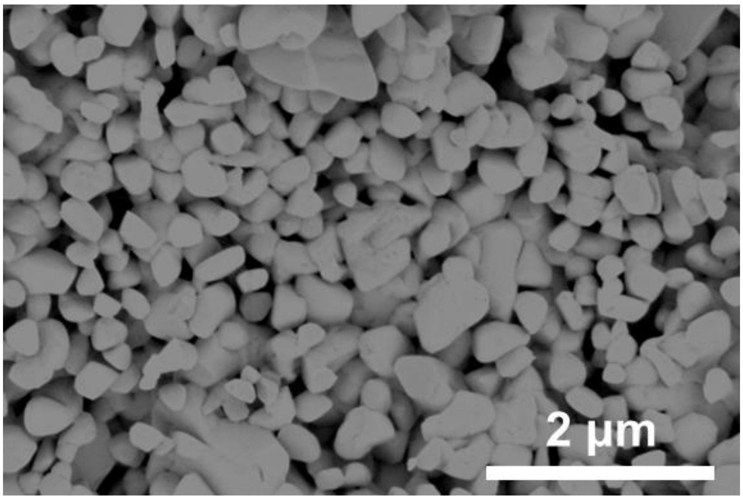

Figure S6. Morphological evolution of CuI in bicarbonate electrolyte. SEM images of a CuI sample immersed in 1 M $KHCO_3$ for 1200 s.

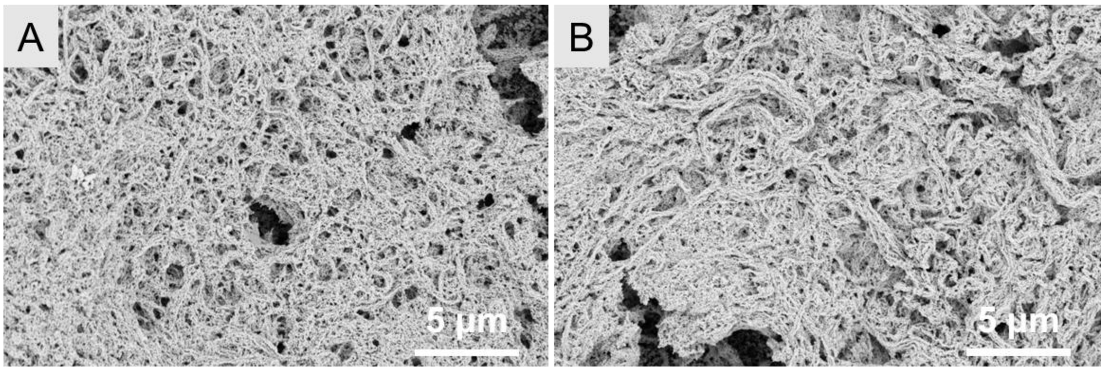

Figure S7. Electrolyte-dependent morphological reconstruction of CuI after electrochemical pre-reduction. SEM images of CuI samples after electrochemical pre-reduction in (A) 0.1 M $KHCO_3$ and (B) 0.1 M KOH.

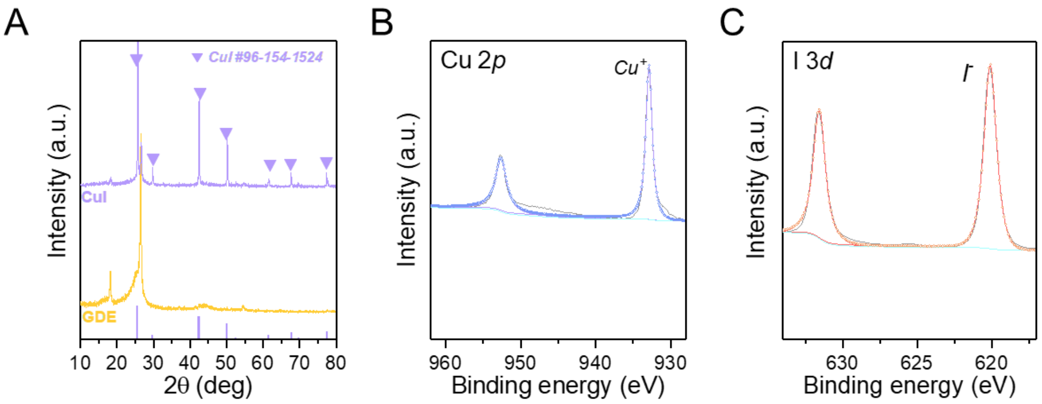

Figure S8. Structural identification and surface chemical state analysis of CuI. (A) X-ray diffractograms of CuI and GDE substrate. High-resolution XPS spectra of Cu 2*p* (B) and I 3*d* (C) of CuI.

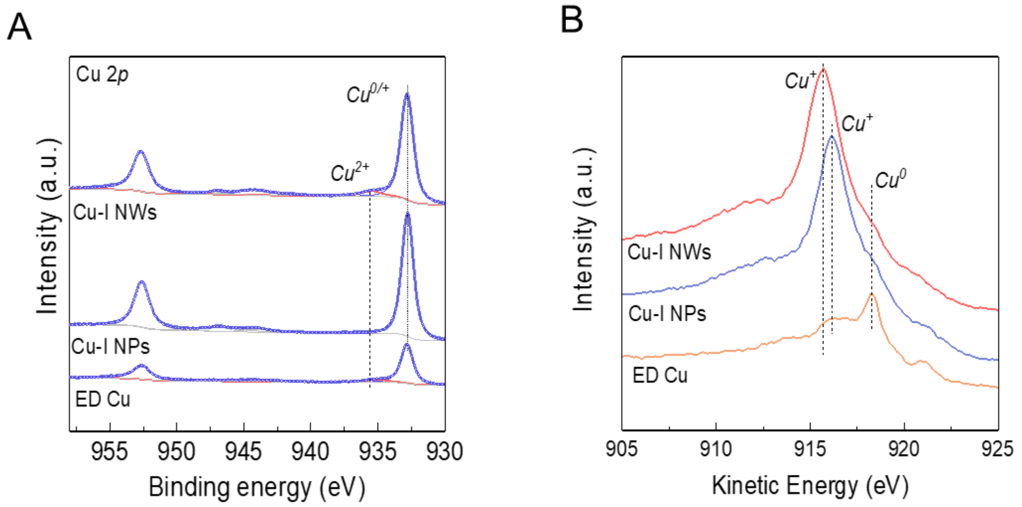

Figure S9. Comparative surface chemical state analysis of ED Cu, Cu-I NPs, and Cu-I NWs. High-resolution XPS spectra of Cu 2*p* (A) and Cu LMM Auger spectra (B) of ED Cu, Cu-I NPs and Cu-I NWs.

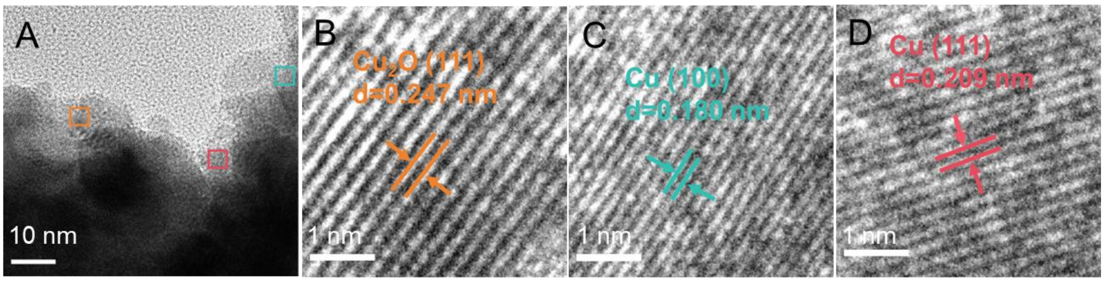

Figure S10. Atomic-scale structural identification of Cu-I NPs. (A) High-resolution transmission electron microscopy image of Cu-I NPs. Lattice fringes corresponding to the (111) facet of Cu_2O (B), the (100) facet (C) and (111) facet (D) of Cu.

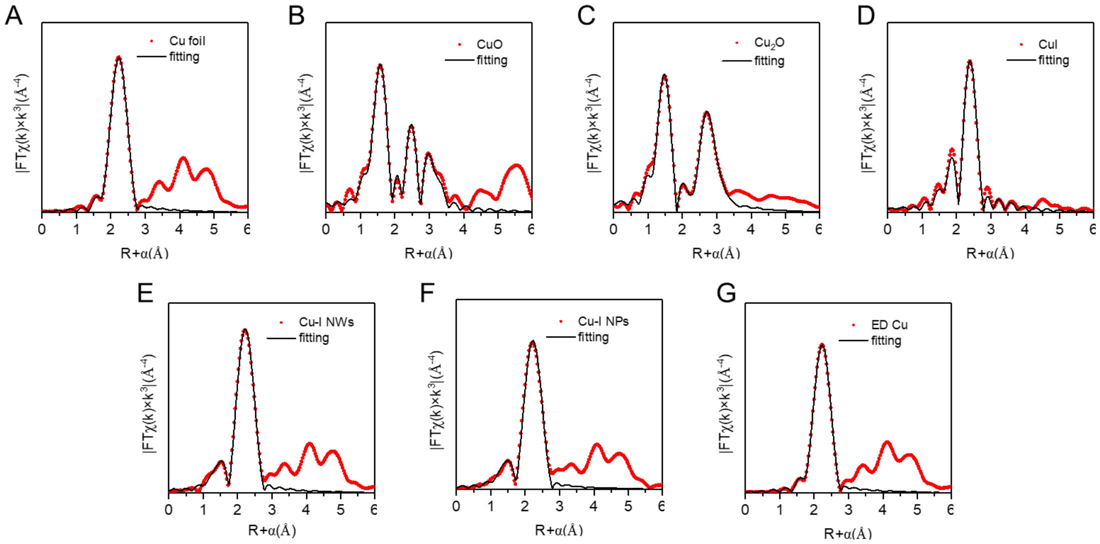

Figure S11. Comparative local coordination analysis of Cu-based samples by EXAFS fitting. R-space fitting plot of the EXAFS spectra of (A) Cu foil, (B) CuO, (C) Cu_2O, (D) CuI, (E) Cu-I NWs, (F) Cu-I NPs and (G) ED Cu.

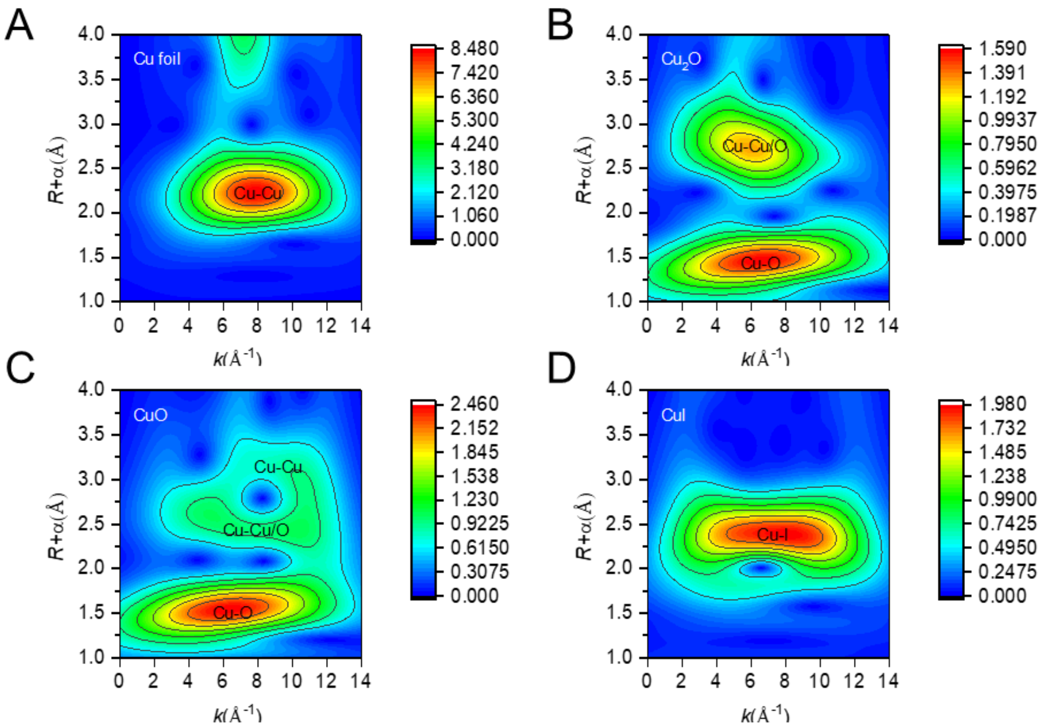

Figure S12. Wavelet transform analysis of local coordination environments in Cu-based materials. Wavelet Transform analysis of the EXAFS data for (A) Cu foil, (B) Cu_2O, (C) CuO and (D) CuI, respectively.

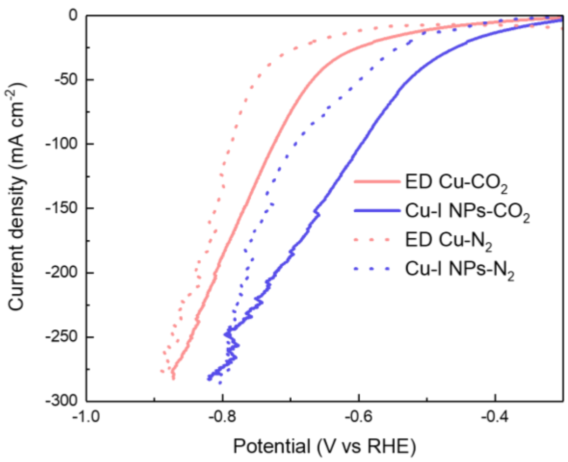

Figure S13. Electrochemical CO_2 reduction performance of ED Cu and Cu–I NPs. Representative Linear sweep voltammograms of ED Cu and Cu-I NPs catalysts in 1 M KOH electrolyte with CO_2 and N_2 gas flow. The curves were recorded at a scan rate of 5 mV s^{-1}.

.

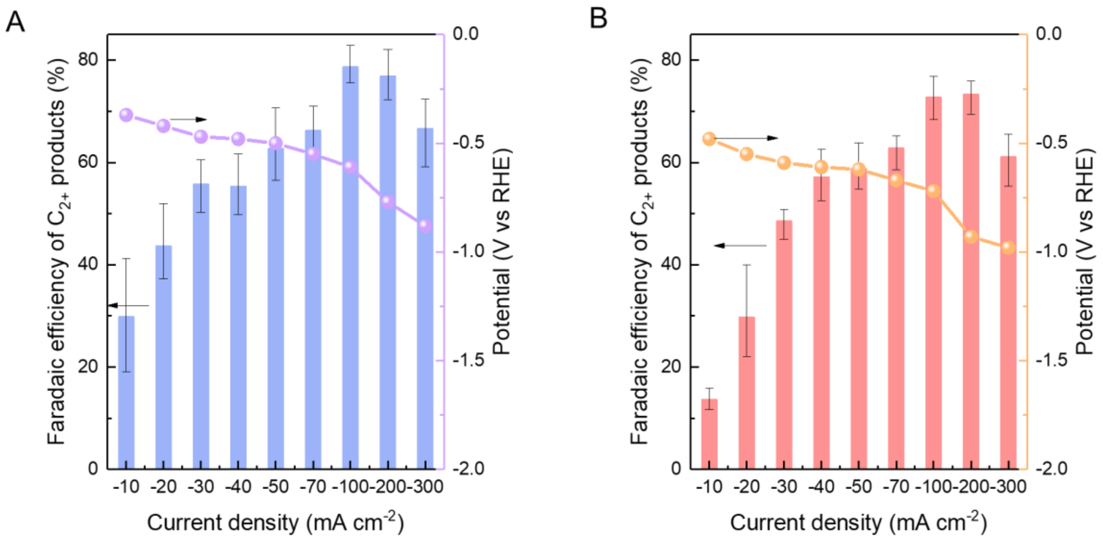

Figure S14. Electrocatalytic performance and C_{2+} product selectivity of Cu-I NPs and ED Cu. FE of CO_2 reduction to multi-carbon products and corresponding potential as a function of current density for (A) Cu-I NPs and (B) ED Cu catalysts**.**

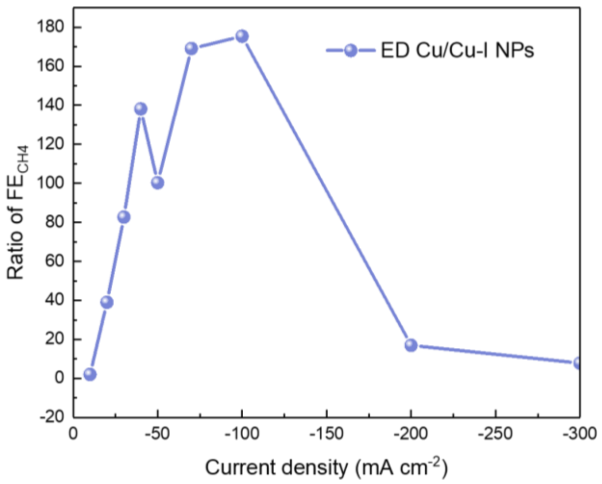

Figure S15. Comparative methane selectivity of ED Cu and Cu-I NPs as a function of current density. The ratio of FE for methane production on ED Cu relative to Cu-I NPs at various applied current densities.

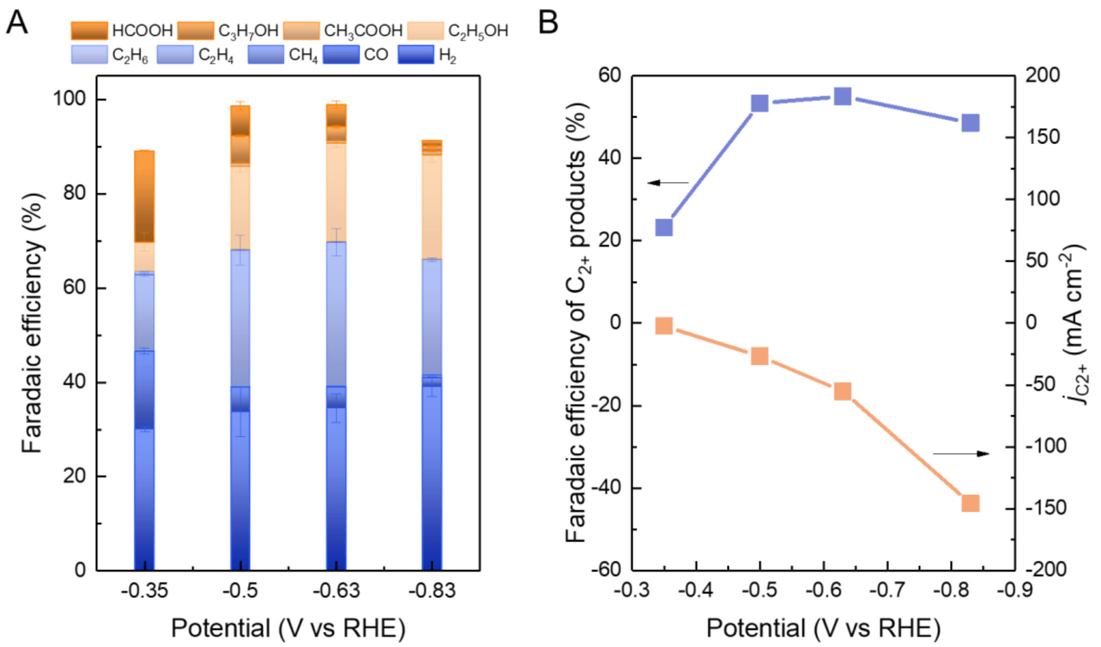

Figure S16. CO_2 reduction performance of Cu-I NWs. (A) FE of CO_2 reduction and (B) corresponding potential as a function of C_{2+} partial current density and FE of C_{2+} products.

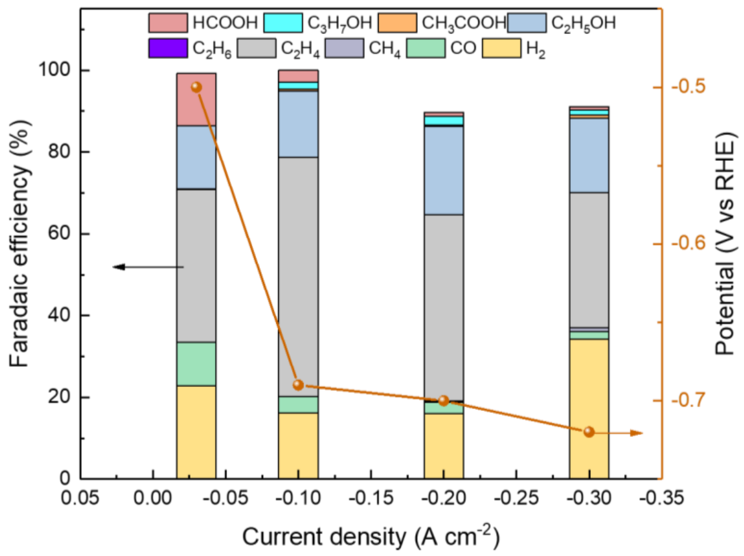

Figure S17. **CO_2 reduction performance of $Cu(OH)_2$ formed by prolonged operation of CuI in 1 M KOH.** FE of CO_2 reduction and corresponding potential as a function of applied current density.

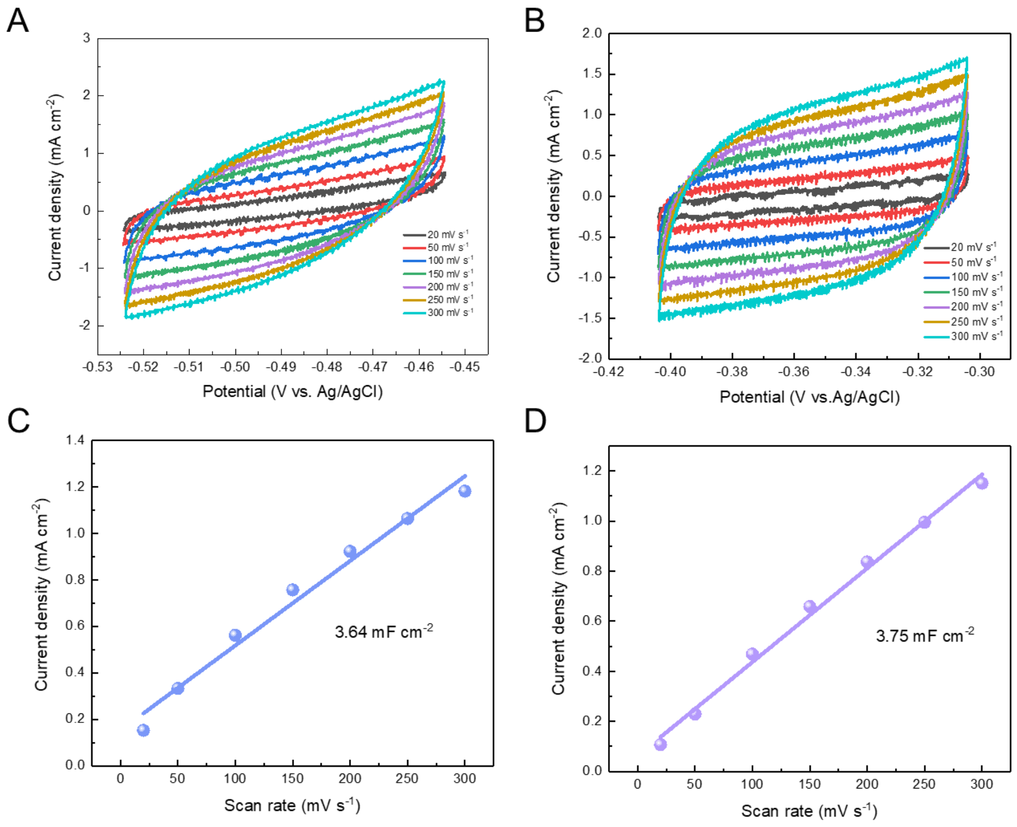

Figure S18. Electrochemical double-layer capacitance and surface area analysis of ED Cu and Cu-I NPs. Representative cyclic voltammetry (CV) curves of (A) ED Cu and (B) Cu-I NPs catalysts measured within the non-faradaic region at sweep rates of 20, 50, 100, 150, 200, 250 and 300 mV s^{-1}. Measurements were conducted in a flow cell with N_2 and 1 M KOH electrolyte supplied to the cathodic gas and liquid chambers. Corresponding current densities at various scan rates for (C) ED Cu and (D) Cu-I NPs, with linear fits used to determine the electrochemical double-layer capacitance (C_{dl}) of each catalyst.

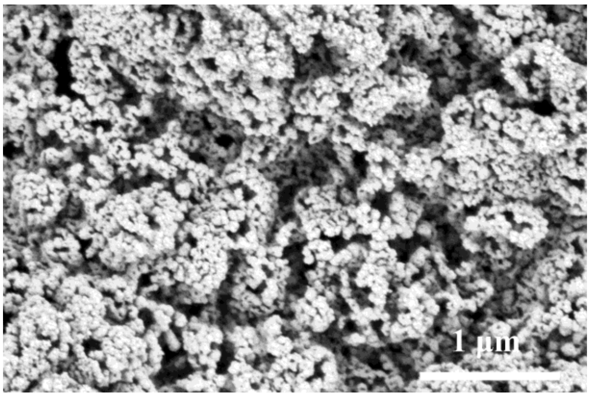

Figure S19. Morphological stability of Cu-I NPs after prolonged CO_2 electrolysis. SEM images of as-synthesized Cu-I NPs after CO_2 electrolysis at -100 mA cm^{-2} for 60 h.

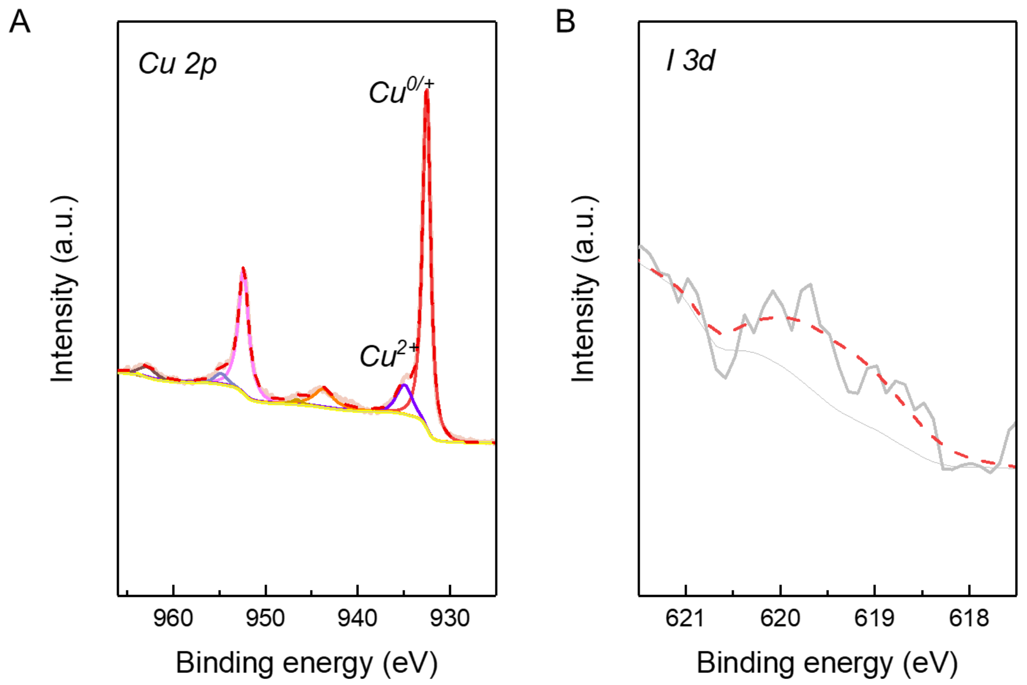

Figure S20. Surface chemical state of Cu-I NPs after prolonged CO_2 electrolysis. XPS spectra of as-synthesized Cu-I NPs showing (A) Cu *2p* and (B) I *3d* regions after CO_2 electrolysis at -100 mA cm^{-2} for 60 h.

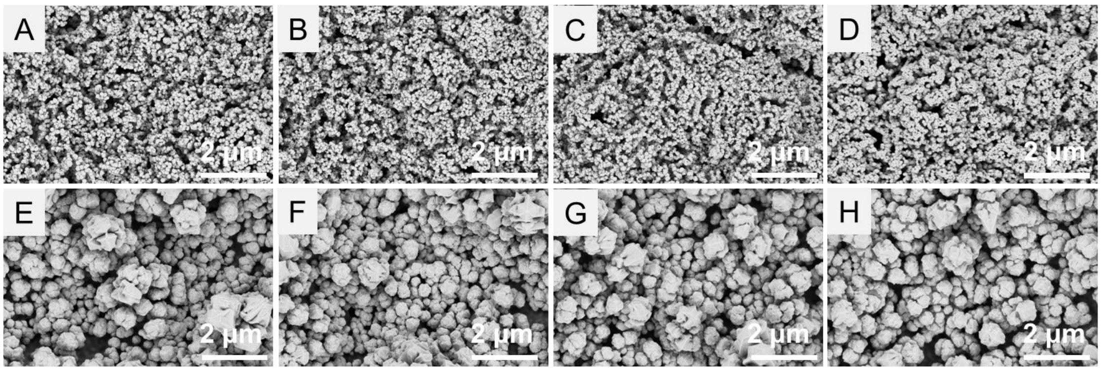

Figure S21. Current-dependent morphological evolution of Cu-I NPs and ED Cu after CO_2 electrolysis. SEM images of as-synthesized Cu-I NPs catalysts after CO_2 electrolysis at different current densities of (A) -30 mA cm^{-2}, (B) -100 mA cm^{-2}, (C) -200 mA cm^{-2}, (D) -300 mA cm^{-2}; SEM images of ED Cu catalysts after CO_2 electrolysis at E) -30 mA cm^{-2}, (F) -100 mA cm^{-2}, (G) -200 mA cm^{-2}, (H) -300 mA cm^{-2}.

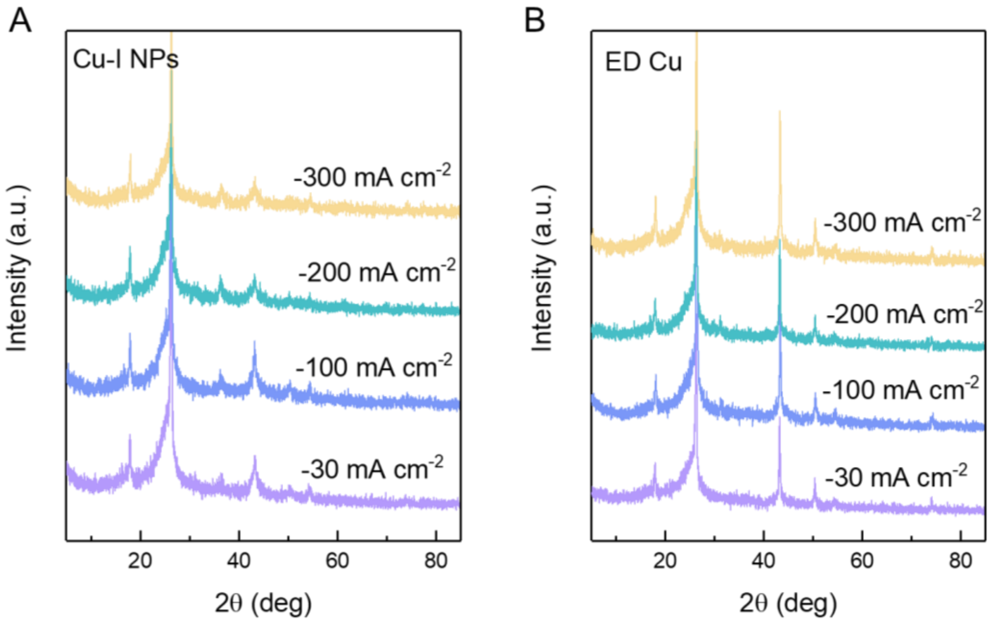

Figure S22. Phase evolution of Cu-I NPs and ED Cu after CO_2 electrolysis at varying current densities. XRD patterns of (A) as-synthesized Cu-I NPs and (B) ED Cu catalysts after CO_2 electrolysis at different current densities.

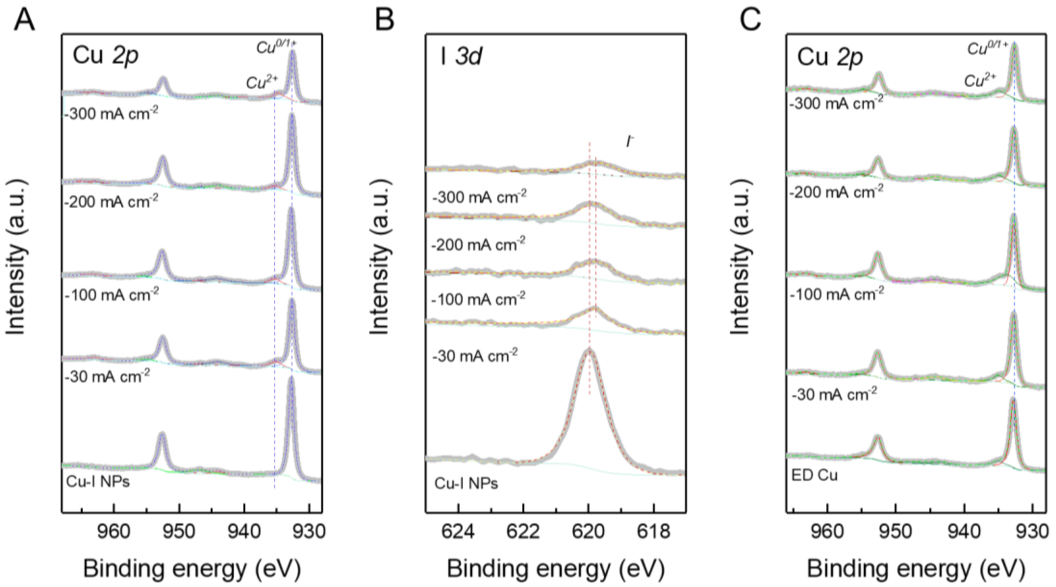

Figure S23. Surface chemical state analysis of Cu-I NPs and ED Cu after CO_2 electrolysis at varying current densities. XPS spectra of as-synthesized Cu-I NPs showing (A) Cu *2p* and (B) I *3d* regions, and (C) Cu *2p* spectra of ED Cu catalysts after CO_2 electrolysis at various current densities.

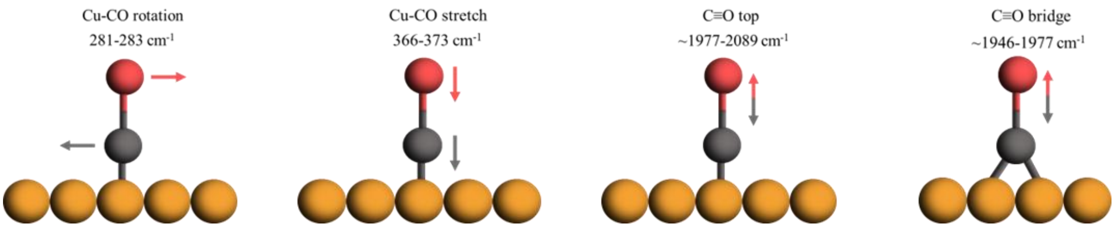

Figure S24. Schematic of CO adsorption behavior and corresponding Raman vibrational signatures. Schematic illustration of CO adsorption behavior and corresponding vibrational bands observed in Raman spectroscopy.

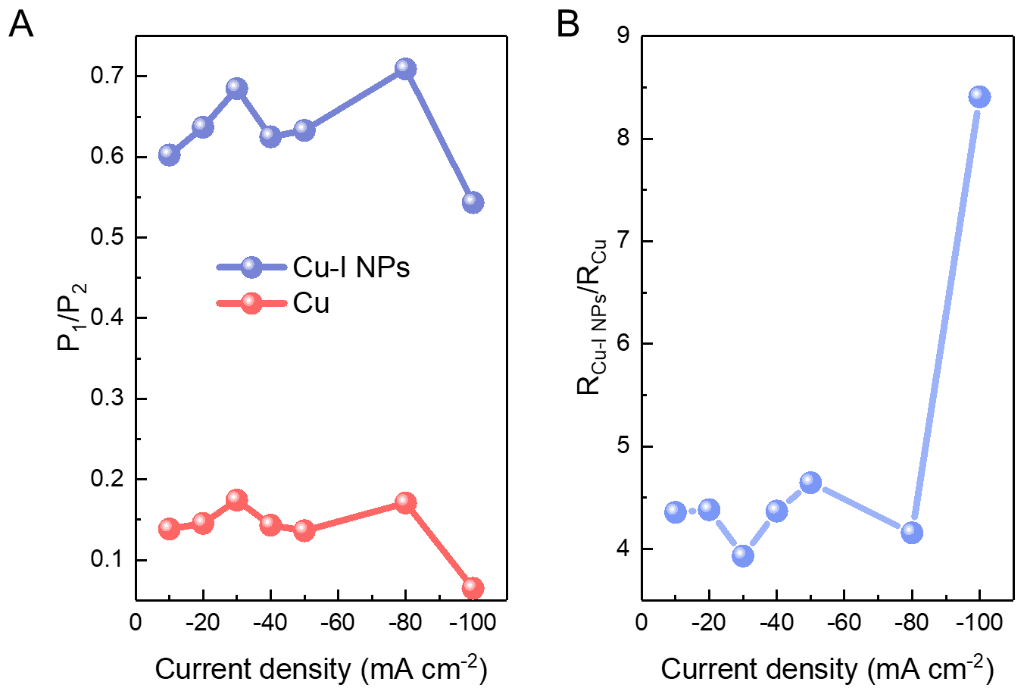

Figure S25. Raman peak area ratio analysis of Cu-I NPs and ED Cu. (A) Ratio of the integrated areas of Raman peaks at 281-283 cm^{-1} (P_1) and 366-373 cm^{-1} (P_2) for Cu-I NPs and ED Cu. (B) Comparative analysis of the peak area ratios from (a) between Cu-I NPs and ED Cu.

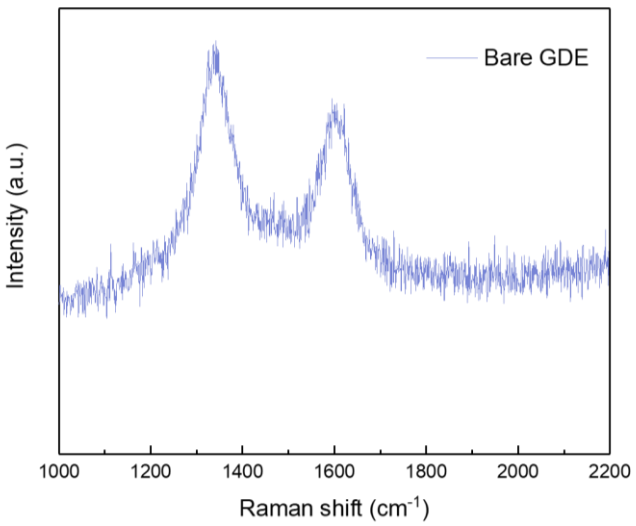

Figure S26. Raman spectrum of the bare GDE under open-circuit conditions. The Raman spectrum of the bare GDE confirming the positions of the D and G bands of the carbon substrate in the absence of electrochemical reaction.

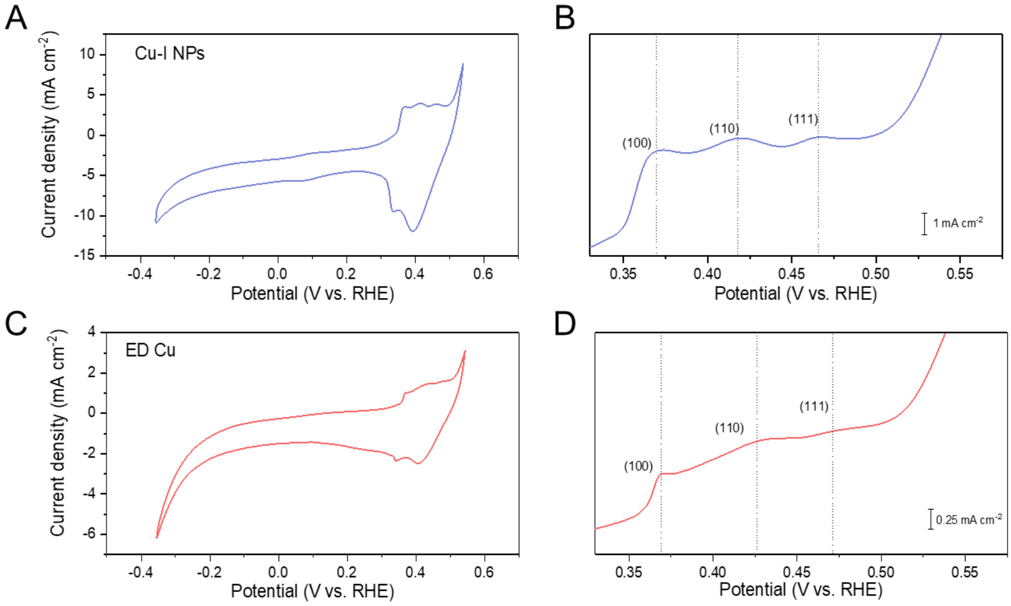

Figure S27. OH^- adsorption on the catalysts was examined through cyclic voltammetry. The voltammograms were obtained for pre-reduced (A and B) Cu-I NPs and (C and D) ED Cu in a 1 M KOH solution under a nitrogen atmosphere. Panels (B) and (D) present detailed views of the anodic peaks, revealing OH^- adsorption on the Cu(100), Cu(110), and Cu(111) facets. The measurements were conducted at a scan rate of 100 mV s^{-1}.

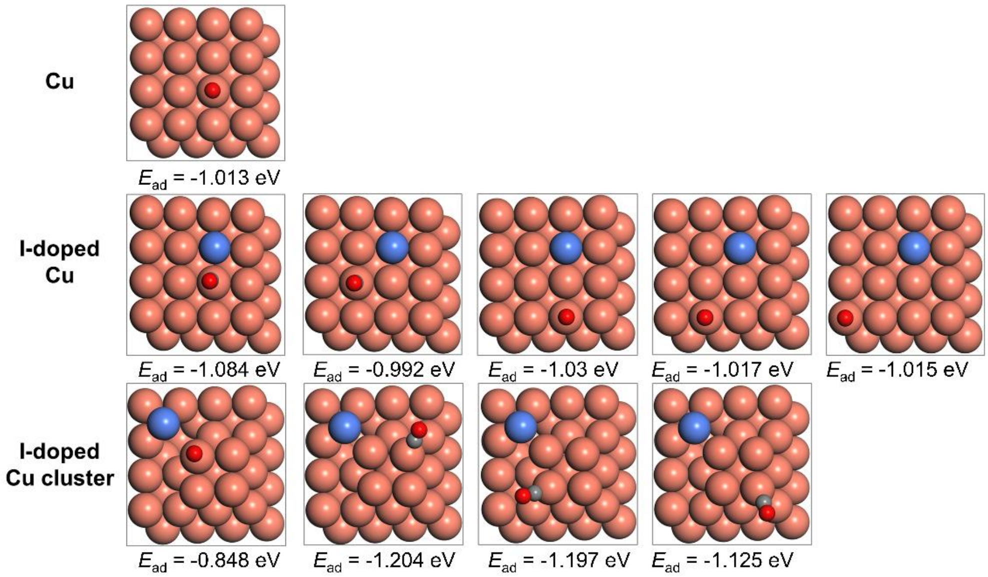

Figure S28. Optimized geometric configurations of a single CO molecule adsorbed on the three constructed theoretical models. To rigorously determine the most thermodynamically stable geometries, various non-equivalent adsorption sites were systematically evaluated for each model. Red, grey, orange and blue balls stand for O, C, Cu and I atoms, respectively.

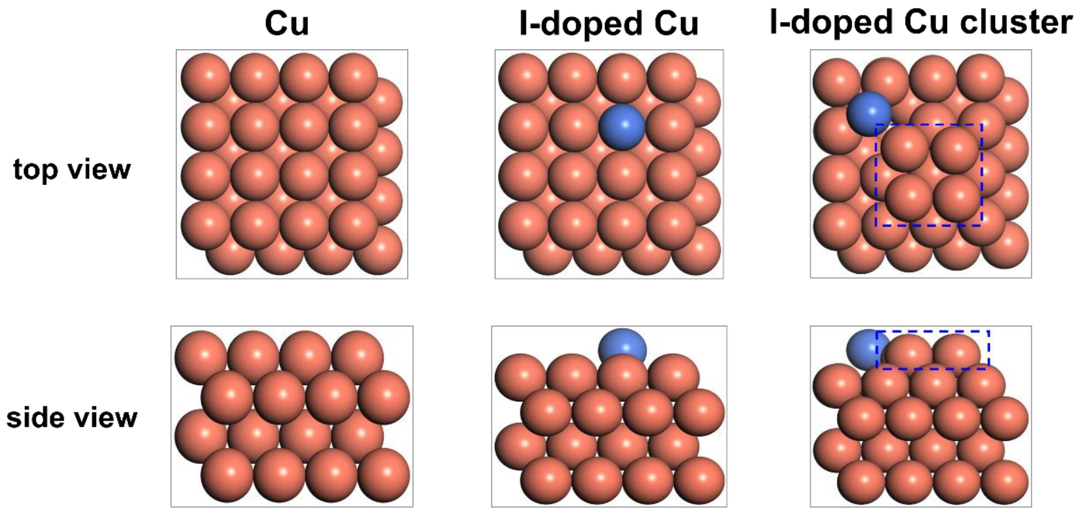

Figure S29. Atomic-level geometries of catalyst models on the Cu(100) facet. Top and side views of geometries on the Cu(100) facet of three catalyst model. Orange and blue balls stand for Cu and I atoms, respectively. The cluster structure on the Cu surface is highlighted by a blue dashed box.

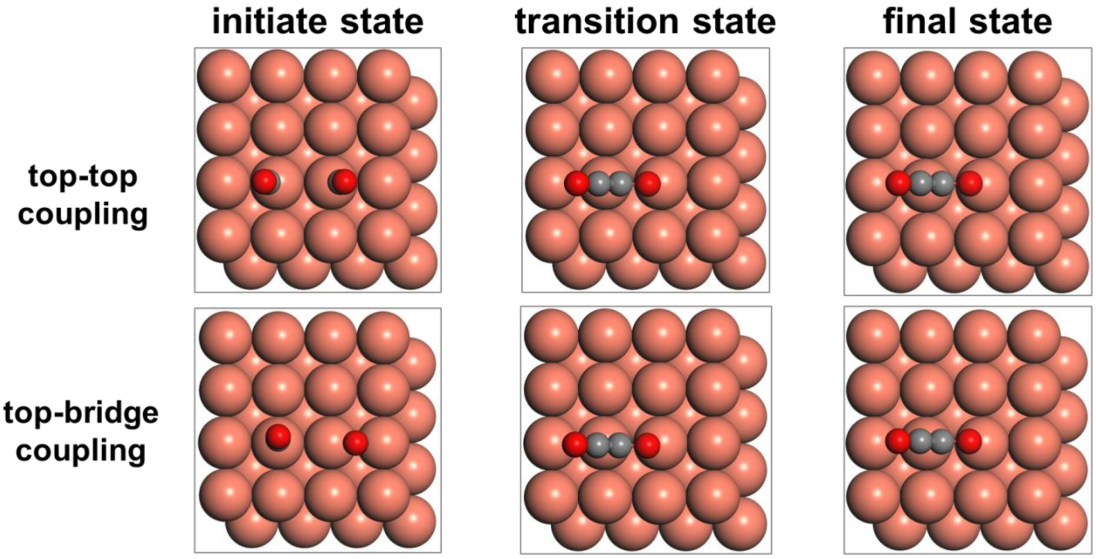

Figure S30. Atomic geometries of C-C dimerization on the Cu(100) facet. Top views of the geometries of top-top and top-bridge for C-C dimerization on the Cu(100) facet of Cu. Red, grey and orange balls stand for O, C and Cu atoms, respectively.

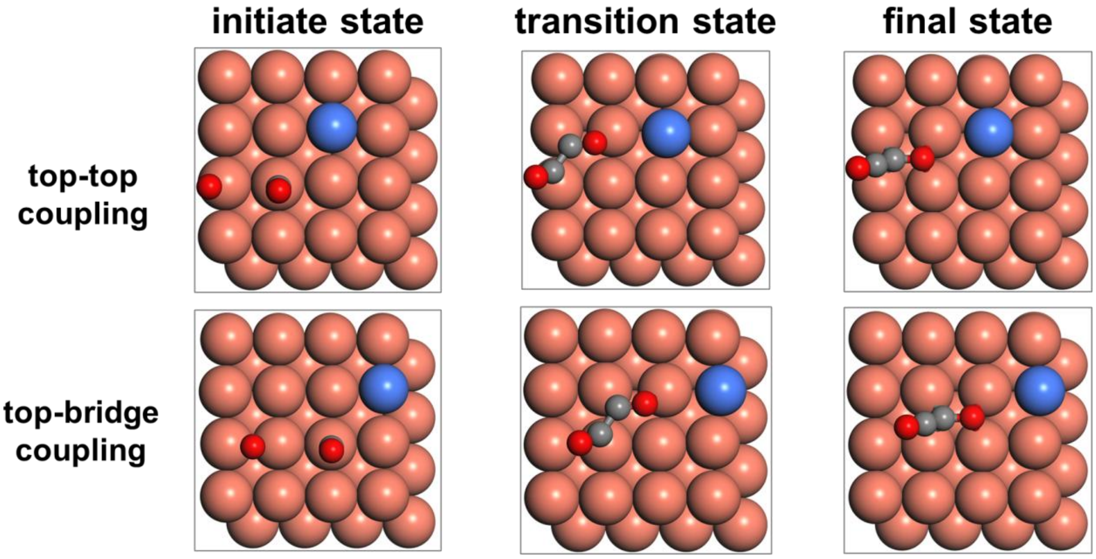

Figure S31. Atomic geometries of C-C dimerization on the I-doped Cu(100) facet. Top views of the geometries of top-top and top-bridge for C-C dimerization on the Cu(100) facet of I-doped Cu. Red, grey, orange and blue balls stand for O, C, Cu and I atoms, respectively.

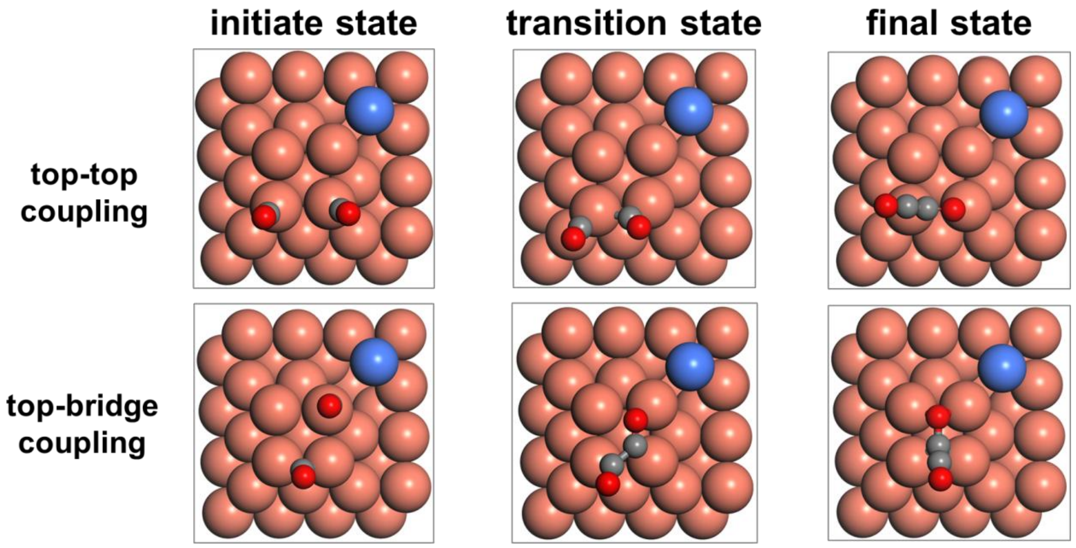

Figure S32. Atomic geometries of C-C dimerization on I-doped Cu clusters on the Cu(100) facet. Top views of the geometries of top-top and top-bridge for C-C dimerization on the Cu(100) facet of I-doped Cu cluster. Red, grey, orange and blue balls stand for O, C, Cu and I atoms, respectively.

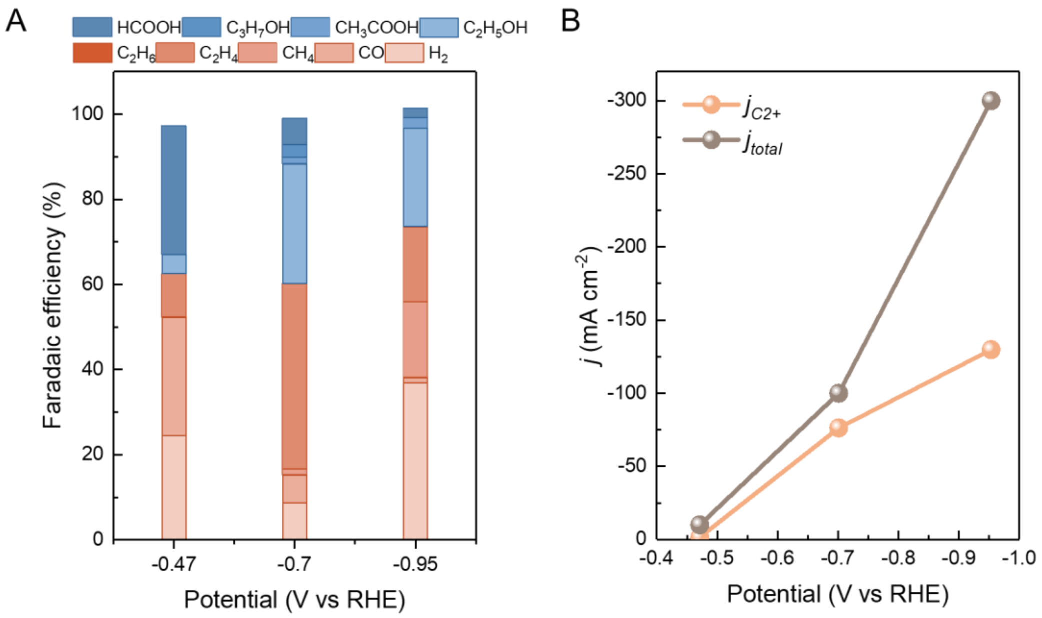

Figure 33. CO_2 reduction performance of Cu NPs. (A) FE of CO_2 reduction and (B) corresponding potential as a function of C_{2+} partial current density and total current density.

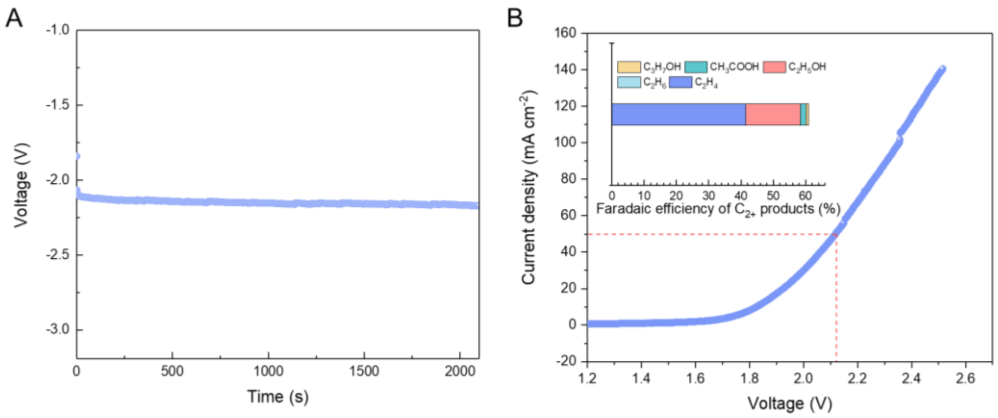

Figure S34. The electrochemical performance in a two-electrode system composed of Cu-I NPs and NiFe layered double hydroxide (LDH) measured. (A) Cell voltage of the system at a current density of -52 mA cm^{-2}. (B) The LSV curves in two-electrode system with the 0.36 cm^2 area of the catalyst and insert figure of (B) corresponding Faradaic efficiencies of C_{2+} products at a current density of -52 mA cm^{-2}.

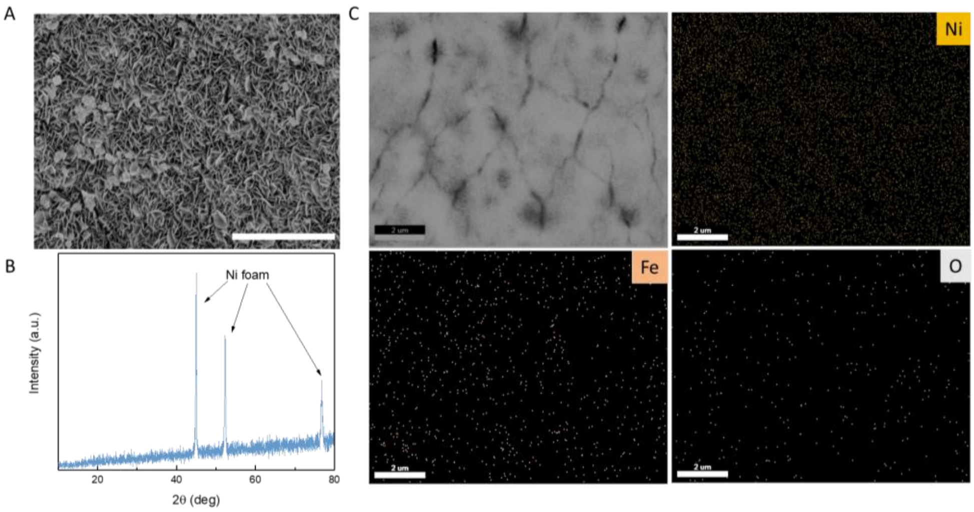

Figure S35. Structural characteristics of NiFe LDH. (A) SEM image of NiFe LDH. (B) XRD pattern of NiFe LDH. (C) EDX elemental mapping of NiFe LDH. Scale bar: 4 μm for A, 2 μm for C.

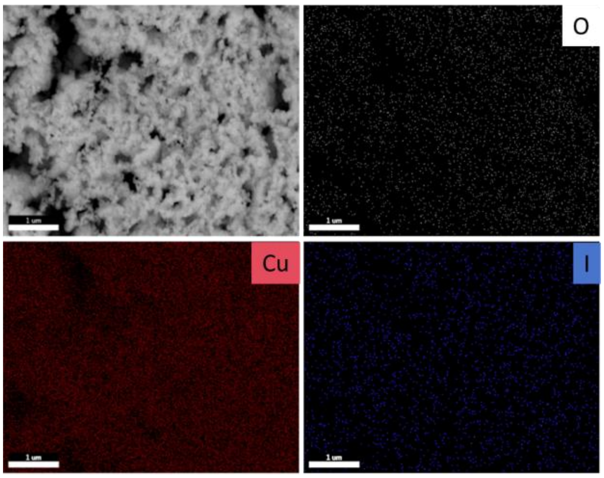

Figure S36. **EDX mapping of Cu-I NPs after extended PV-EC operation.** Elemental distribution maps of Cu-I NPs after 120 min operation in the solar-driven PV-EC system, obtained by EDX analysis. Scale bar: 1 μm.

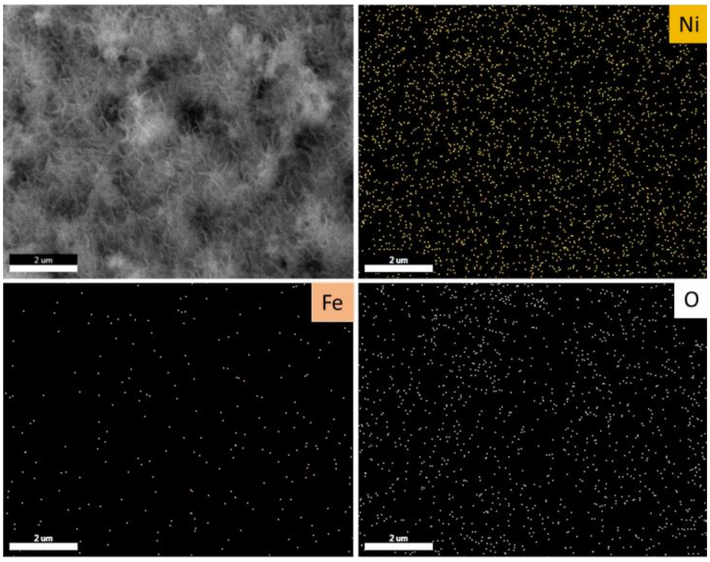

Figure S37. **EDX mapping of NiFe LDH after extended PV-EC operation.** Elemental distribution maps of NiFe LDH after 120 min operation in the solar-driven PV-EC system, obtained by EDX analysis. Scale bar: 2 μm.

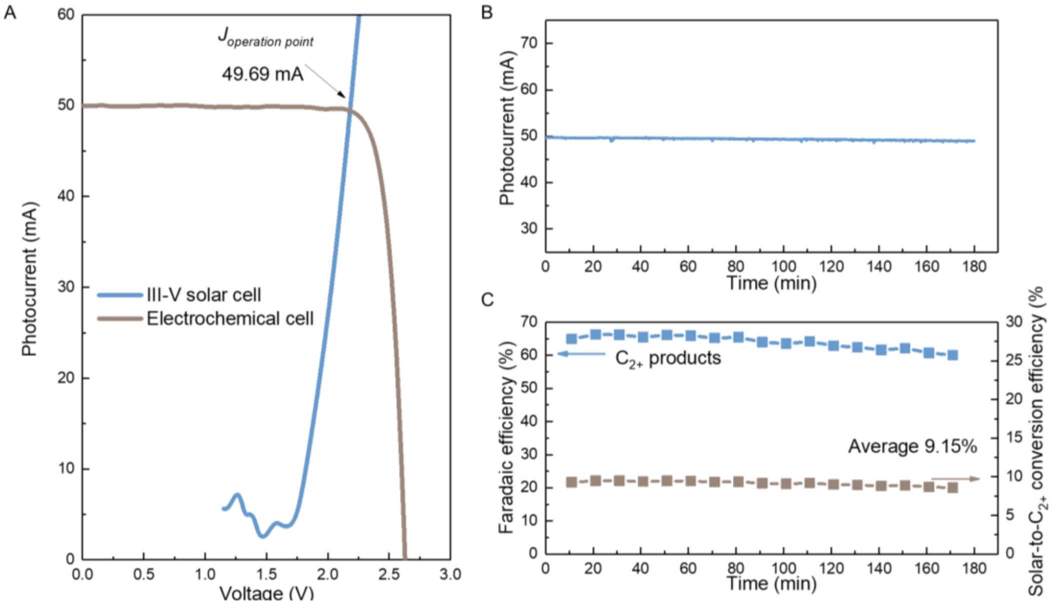

Figure S38. **Solar-driven conversion of CO_2 into multi-carbon products via an integrated PV-EC system.** (A) Linear sweep voltammetry profiles of the photovoltaic module and the electrochemical cell. (B) Operating current density of the self-powered PV-EC system under simulated AM 1.5 G solar irradiation. (C) Faradaic efficiencies for multi-carbon products and the corresponding solar-to-multicarbons energy conversion efficiencies achieved by the unassisted PV-EC system under AM 1.5 G illumination. Specifically, four of III-V cells were connected in parallel to supply the required current. The active irradiation area of a single solar cell is 0.9801 cm^2.

Table S1. The Cu K-post-edge XAS fitting results for Cu foil, Cu_2O, CuO, CuI, ED Cu, Cu-I NPs and Cu-I NWs catalysts.

Sample	Shell	*CN*	*R(Å)*	$\sigma^2(Å^2)$	$\Delta E_0(eV)$	*R* factor
Cu foil	Cu-Cu	12*	2.54±0.01	0.0085±0.0004	4.0±0.2	0.0027
Cu_2O	Cu-O	2*	1.85±0.01	0.0026±0.0007	7.8±0.4	0.0054
	Cu-Cu	12*	3.03±0.01	0.0204±0.0009	8.7±0.3	
	Cu-O	6*	3.56±0.01	0.0152±0.0026		
CuO	Cu-O	4.0±0.2	1.95±0.01	0.0027±0.0006	7.9±0.2	0.0021
	Cu-O	2.6±0.4	2.76±0.01			
	Cu-Cu	1.9±0.3	2.85±0.01	0.0034±0.0009	-8.4±0.9	
	Cu-Cu	2.1±0.3	3.37±0.01			
CuI	Cu-I	4.0±0.1	2.58±0.01	0.0102±0.0004	0.2±0.2	0.0044
ED Cu	Cu-Cu	11.6±0.5	2.54±0.01	0.0086±0.0004	4.0±0.2	0.0022
Cu-I NPs	Cu-O	0.7±0.2	1.84±0.01	0.0017±0.0042	7.0±2.3	0.0080
	Cu-Cu/I	7.2±0.8	2.54±0.01	0.0091±0.0010	4.3±0.5	
Cu-I NWs	Cu-O	0.7±0.2	1.85±0.01	0.0025±0.0037	7.8±1.7	0.0051
	Cu-Cu/I	5.6±0.5	2.54±0.01	0.0083±0.0008	4.1±0.4	

CN, coordination number; *R*, the distance to the neighboring atom; σ^2, the Mean Square Relative Displacement (MSRD); ΔE_0, inner potential correction; *R* factor indicates the goodness of the fit.

Table S2. Faradaic efficiencies of products obtained from CO_2 reduction on Cu-I NPs at different current densities with applied potential.

Potential (V vs RHE)	J_{total} (mA cm^{-2})	H_2	CO	CH_4	C_2H_4	C_2H_6	C_2H_5OH	CH_3COOH	C_3H_7OH	HCCOH	Total
-0.37	-10	20.04	24.19	0.0069	19.19	0.28	7.02	3.33	0	17.16	91.22
-0.42	-20	20.71	14.89	0.0042	28.11	0.21	8.81	1.08	5.51	13.43	92.75
-0.47	-30	17.94	10.11	0.0044	35.22	0.20	13.38	1.94	5.01	13.01	96.81
-0.48	-40	17.41	8.71	0.0064	38.47	0.15	11.56	1.17	3.98	12.37	93.83
-0.5	-50	15.22	7.41	0.0099	42.96	0.11	16.15	0.74	2.74	9.57	94.90
-0.55	-70	14.69	4.49	0.015	45.87	0.08	16.47	0.72	3.12	7.54	93.00
-0.61	-100	14.51	3.84	0.015	48.85	0.05	24.59	0.53	4.68	4.44	101.51
-0.77	-200	20.43	3.05	0.35	50.12	0.0094	23.60	0.41	2.73	1.46	102.16
-0.88	-300	31.58	2.58	1.22	37.39	0.005	26.98	1.05	1.25	1.18	103.24

Table S3. Faradaic efficiencies of products obtained from CO_2 reduction on ED Cu at different current densities with applied potential.

Potential (V vs RHE)	J_{total} (mA cm^{-2})	H_2	CO	CH_4	C_2H_4	C_2H_6	C_2H_5OH	CH_3COOH	C_3H_7OH	HCCOH	Total
-0.48	-10	21.10	33.16	0.014	11.77	0.030	1.08	0.37	0	23.77	91.29
-0.55	-20	17.55	27.29	0.16	22.17	0.030	5.27	0.044	2.14	13.69	88.34
-0.59	-30	15.70	20.89	0.37	29.41	0.031	13.06	1.13	3.79	12.94	97.32
-0.61	-40	13.41	16.92	0.89	36.71	0.022	14.58	0.57	4.71	9.17	96.98
-0.62	-50	12.20	16.25	0.99	39.73	0.023	15.25	0.63	2.50	6.67	94.24
-0.67	-70	10.31	11.59	2.60	40.37	0.016	17.81	1.15	2.33	6.57	92.75
-0.72	-100	9.60	7.81	2.70	47.29	0.014	21.37	1.18	1.75	3.80	95.51
-0.93	-200	17.83	5.88	5.85	40.67	0.009	26.83	2.60	0.62	2.13	102.42
-0.98	-300	27.10	4.88	9.50	29.13	0.008	22.89	4.46	0.18	1.98	100.13

Table S4. Summary of iodine-modified Cu catalysts for multi-carbon product generation, including onset potentials, in alkaline flow cell systems.

Sample	Electrolyte	Onset-Potential (V vs.RHE)	FE_{C2+} (%)	Ref
SCF-50/CuI	1 M KOH	~-0.9	45	32
W-ID-Cu	1 M KOH	-0.7	35	33
I-OD-Cu	1 M KOH	~-0.62	~60	35
I-mediated Cu-based	1 M $KHCO_3$ +0.1 M KI	~-0.57	~2.5	34
I-Cu	1 M KOH	-0.4	~10	31
Cu-I NPs	1 M KOH	-0.37	30	This work

Table S5. The amount of I and Cu in Cu-I NPs after electrochemical tests at current densities of -30, -100, -200, and -300 mA cm^{-2}, as determined by ICP-OES.

**Current density (mA cm^{-2})**	**I (wt%)**	**Cu(wt%)**	**$Ratio_{Cu/I}$**
-30	0.18	2.8	15.56
-100	0.15	2.6	17.33
-200	0.13	2.7	20.77
-300	0.08	1.8	22.50

Table S6. Summary of Raman peaks over ED Cu and Cu-I NPs catalysts for CO_2RR from Fig. 3.

**Sample**	**Raman shift (cm^{-1})**	**Species**
ED Cu and Cu-I NPs	281-283	Cu–C restricted rotation
	363-373	Cu–C stretching vibrations
	537	adsorbed $CuO_x/(OH)_y$ species
	670	OCH vibrational modes
ED Cu	1064	adsorbed carbonate species
ED Cu	1268	-C=O stretching and C-OH vibrations
ED Cu	1334-1356	carboxylate groups ($*CO_2^-$)
ED Cu	1584-1605	carboxylate groups ($*CO_2^-$)
Cu-I NPs	1061	adsorbed carbonate species
Cu-I NPs	1286	-C=O stretching and C-OH vibrations
Cu-I NPs	1540-1609	carboxylate groups ($*CO_2^-$)

Table S7. Charge values for OH^- adsorption on various facets of Cu-I NPs and ED Cu catalysts, estimated from the cyclic voltammograms presented in Supplementary Figure 25. The reference charges for OH^- adsorption on Cu(100), Cu(111), and Cu(110) single crystals are 59, 79, and 27 μC cm^{-2}, respectively *(54)*. The surface area of both Cu-I NPs and ED Cu in the H-cell was measured to be 0.2 cm^2.

**Catalyst**	**Charge of Cu(100) (mC cm^{-2})**	**Charge of Cu(111) (mC cm^{-2})**	**Charge of Cu(110) (mC cm^{-2})**	**Surface area of Cu(100) (cm^{-2})**	**Surface area of Cu(111) (cm^{-2})**	**Surface area of Cu(110) (cm^{-2})**	**Cu(100)/Cu(110)**	**Cu(100)/Cu(111)**
Cu-I NPs	0.019	0.015	0.006	0.064	0.038	0.04	1.60	1.68
ED Cu	0.0003	0.0006	0.0004	0.001	0.0015	0.003	0.33	0.67

Table S8. Energy efficiency and overall reaction performance for multi-carbon product generation in solar-driven electrolyzers.

**Reduction product**	**Overall reaction**	**Equilibrium potential (V)**	**Faradaic efficiency (%)**	**Solar to fuel conversion efficiency (%)**
Ethane	$4CO_2 + 6H_2O \rightarrow 2C_2H_6 + 7O_2$	1.09	0.15	0.02
Acetate	$2CO_2 + 2H_2O \rightarrow CH_3COO^- + H^+ + 2O_2$	1.15	1.68	0.24
Ethylene	$2CO_2 + 2H_2O \rightarrow C_2H_4 + 3O_2$	1.15	34.20	4.85
Ethanol	$2CO_2 + 3H_2O \rightarrow C_2H_5OH + 3O_2$	1.14	25.10	3.53

Table S9. Overview of systems integrating photovoltaic power with electrochemical CO_2 reduction for multi-carbon product synthesis.

No.	S.T.C. *	ST-C_2H_4	Cathode	Anode	Voltage at operation point	Light-harvesting materials	Ref.
1		2.30%(C_2H_4 and C_2H_6)	Dendritic nanostructured CuO	The same as cathode	2.8 V	$FA_{1-x}MA_xPb(I_{1-y}Br_y)_3$ perovskite	57
2	1.50%**		Ag-supported dendritic Cu	IrO_2 nanotube	2.46 V	$CH_3NH_3PBI_3$ perovskite coupling Si photocathode	11
3	2.90% (AM 1.0)	1.50%	Cu_2O derived Cu	IrO_x	3.3 V	p-n^+ Si	56
4	5.60%**	3.00% (C_2H_4 and C_2H_6)	Nanocoral CuAg bimetal	IrO_2 nanotube	2.5 V	III–V/Si tandem solar cell	12
5	5.55%	4.20%	Ag decorated Cu nanowires	IrO_x	2.76 V	$Cs_x(MA_{0.17}FA_{0.83})_{(100-x)}Pb(I_{0.83}Br_{0.17})_3$ perovskite	10
6	~6.00%	4.00%	Cu(100)-rich film	Ni foam	2.41 V	p-n^+ Si	9
7	6.40	4.47	$Cu(OH)_2$-D/CP	Ni foam/NiFe-LDH/NF	2.74 V	Five-junction single-crystal Si photovoltaic	8
8	8.64%	4.85%	Cu-I NPS	NiFe	2.17 V	Perovskite/silicon-Silicon	This work

*The S.T.C calculations include only C_{2+} products.

** The S.T.C calculations include hydrocarbons and oxygenates.

Table S10. Energy efficiency and overall reaction performance for multi-carbon products generation in solar-driven electrolyzers by III-V solar cells.

**Reduction product**	**Overall reaction**	**Equilibrium potential (V)**	**Faradaic efficiency (%)**	**Solar to fuel conversion efficiency (%)**
Ethane	$4CO_2 + 6H_2O \rightarrow 2C_2H_6 + 7O_2$	1.09	0.039	0.005
Acetate	$2CO_2 + 2H_2O \rightarrow CH_3COO^- + H^+ + 2O_2$	1.15	1.64	0.24
Propanol	$6CO_2 + 8H_2O \rightarrow 2C_3H_7OH + 9O_2$	1.02	5.86	0.75
Ethylene	$2CO_2 + 2H_2O \rightarrow C_2H_4 + 3O_2$	1.15	41.44	6.00
Ethanol	$2CO_2 + 3H_2O \rightarrow C_2H_5OH + 3O_2$	1.14	14.97	2.15
